# Supplementary figures and images for: Microtubule-mediated GLUT4 trafficking is disrupted in insulin-resistant skeletal muscle
Source: eLife. 2023 Apr 19;12:e83338. doi: 10.7554/eLife.83338 (PMC10171867; doi:10.7554/eLife.83338)

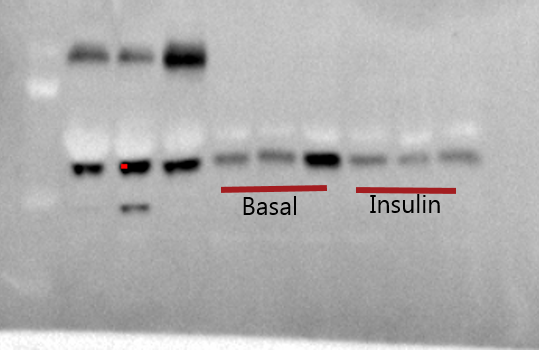

Supplement: Figure 2—figure supplement 1—source data 1. [file elife-83338-fig2-figsupp1-data1.zip › Fig. 2 - supp. 1/Figure 2 - supplement 1 A Akt membrane marked.png]

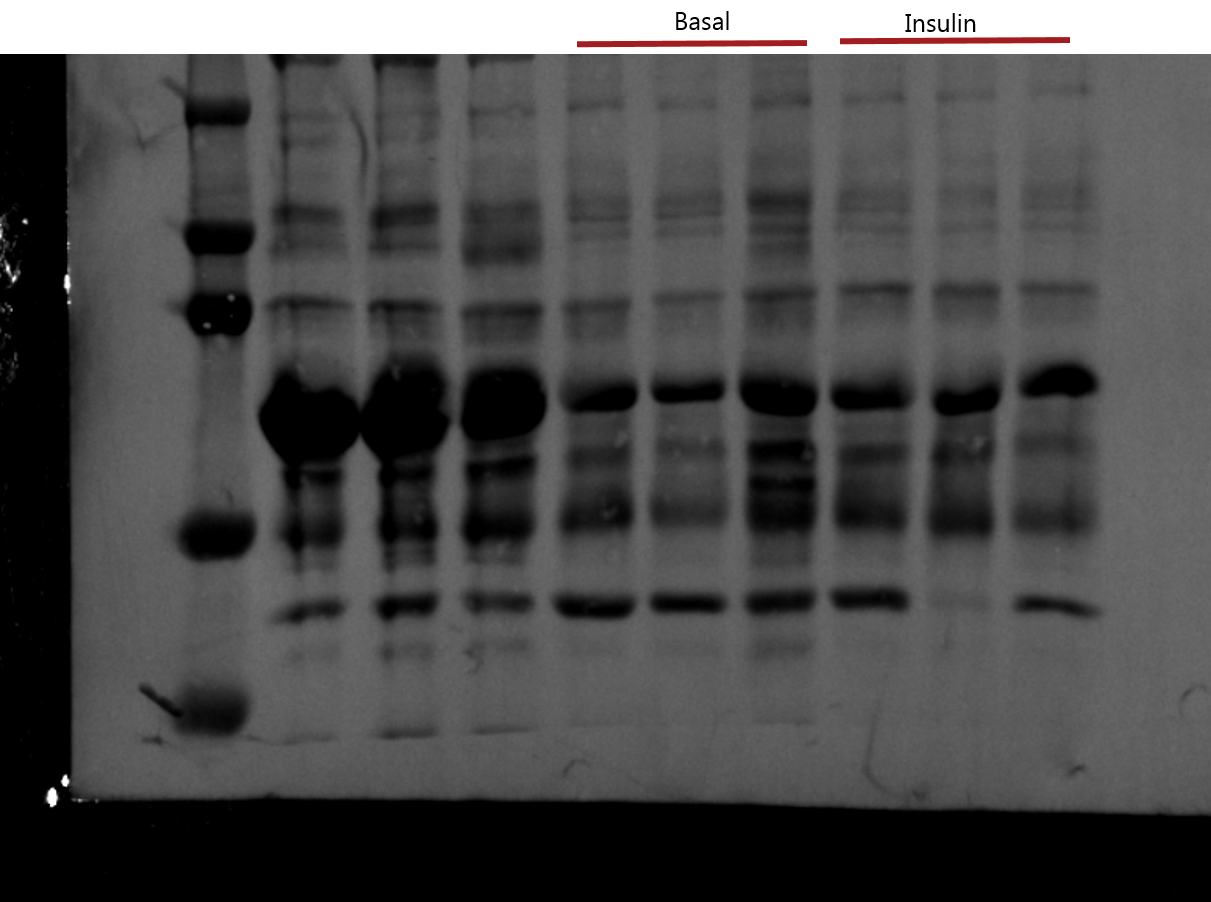

Supplement: Figure 2—figure supplement 1—source data 1. [file elife-83338-fig2-figsupp1-data1.zip › Fig. 2 - supp. 1/Figure 2 - supplement 1 A coomassie membrane marked.png]

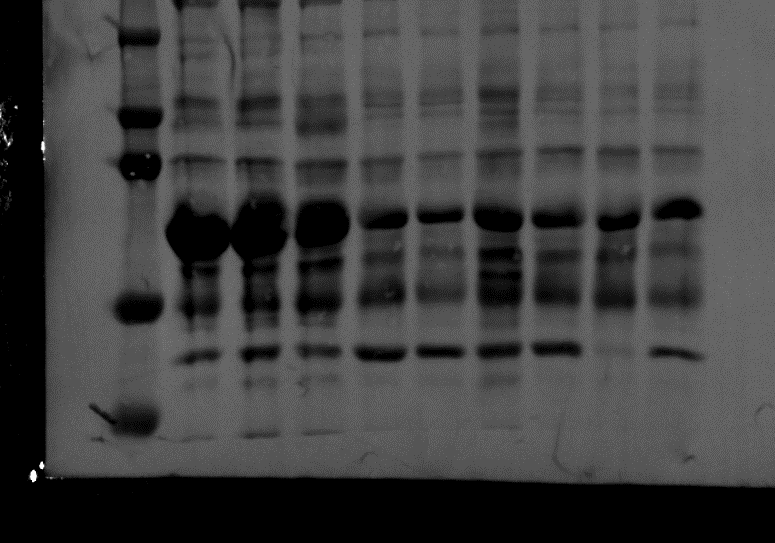

Supplement: Figure 2—figure supplement 1—source data 1. [file elife-83338-fig2-figsupp1-data1.zip › Fig. 2 - supp. 1/Figure 2 - supplement 1 A coomassie membrane.png]

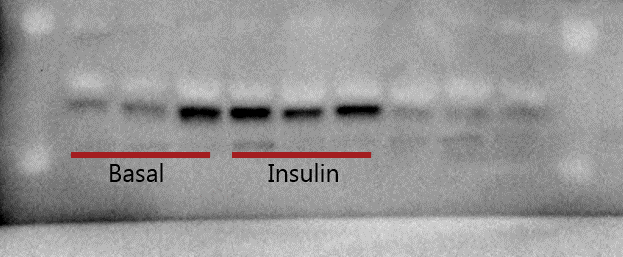

Supplement: Figure 2—figure supplement 1—source data 1. [file elife-83338-fig2-figsupp1-data1.zip › Fig. 2 - supp. 1/Figure 2 - supplement 1 A p-Akt 308 membrane marked.png]

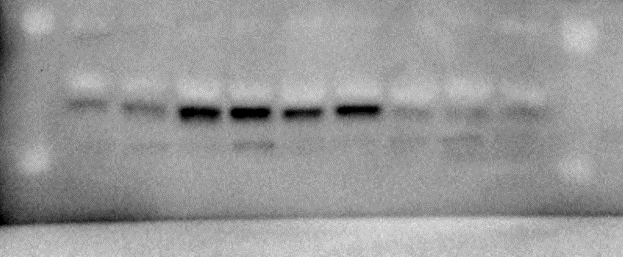

Supplement: Figure 2—figure supplement 1—source data 1. [file elife-83338-fig2-figsupp1-data1.zip › Fig. 2 - supp. 1/Figure 2 - supplement 1 A p-Akt 308 membrane.png]

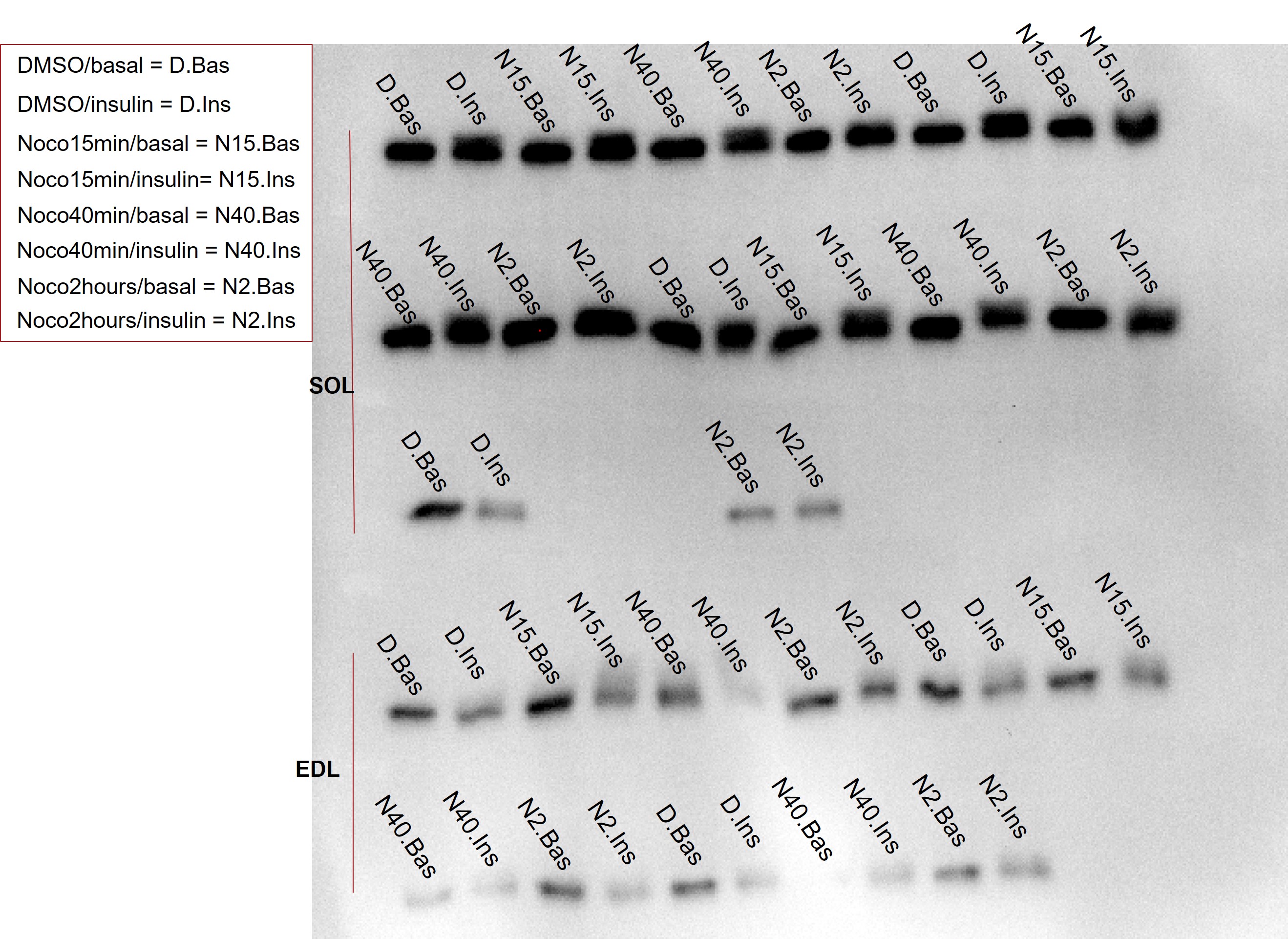

Supplement: Figure 3—figure supplement 1—source data 1. — Data used for quantification of Figure 3—figure supplement 1A, B, F, I and raw unedited blots for Figure 2—figure supplement 1A, B. [file elife-83338-fig3-figsupp1-data1.zip › Figure 3 - supplement 1/Figure 3 - figure supplement 1 A+B Akt2 membrane 1 marked.jpg]

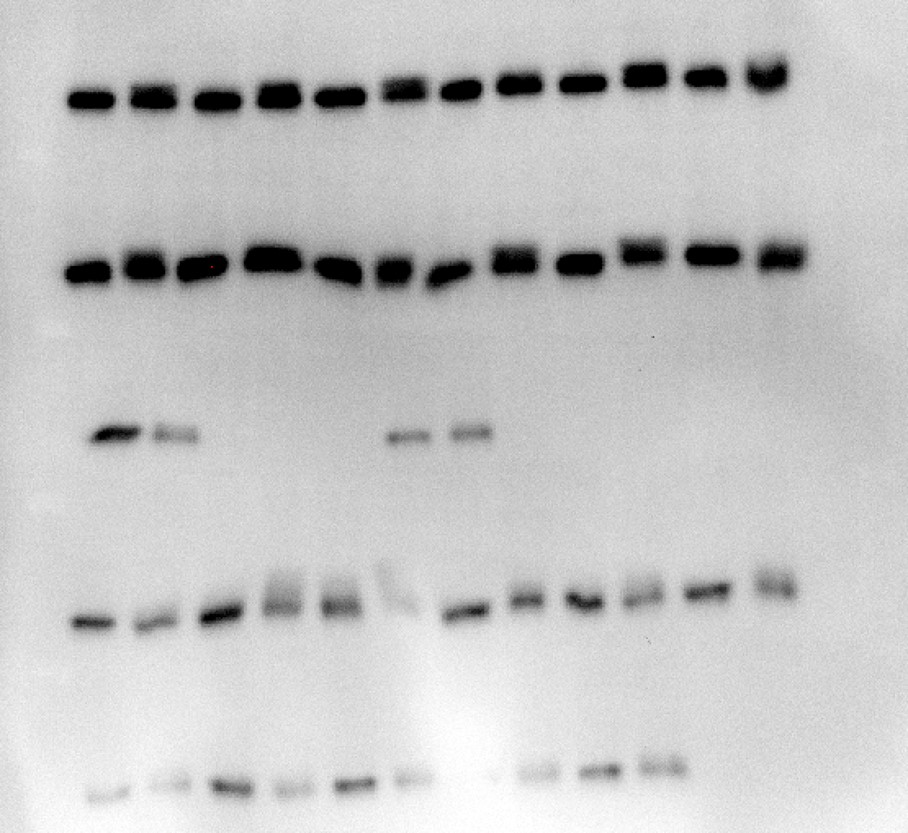

Supplement: Figure 3—figure supplement 1—source data 1. — Data used for quantification of Figure 3—figure supplement 1A, B, F, I and raw unedited blots for Figure 2—figure supplement 1A, B. [file elife-83338-fig3-figsupp1-data1.zip › Figure 3 - supplement 1/Figure 3 - figure supplement 1 A+B Akt2 membrane 1.jpg]

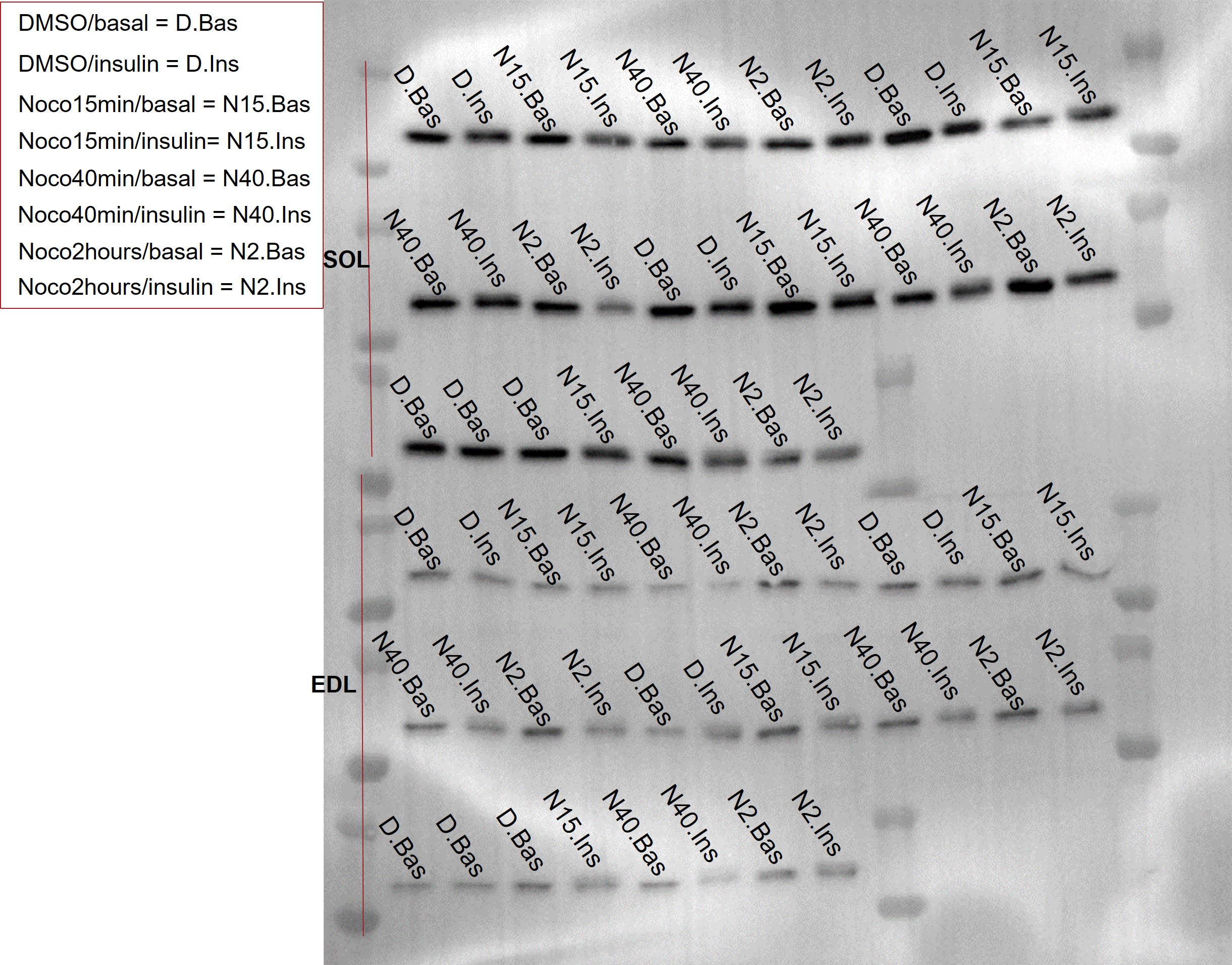

Supplement: Figure 3—figure supplement 1—source data 1. — Data used for quantification of Figure 3—figure supplement 1A, B, F, I and raw unedited blots for Figure 2—figure supplement 1A, B. [file elife-83338-fig3-figsupp1-data1.zip › Figure 3 - supplement 1/Figure 3 - figure supplement 1 A+B Akt2 membrane 2 marked.jpg]

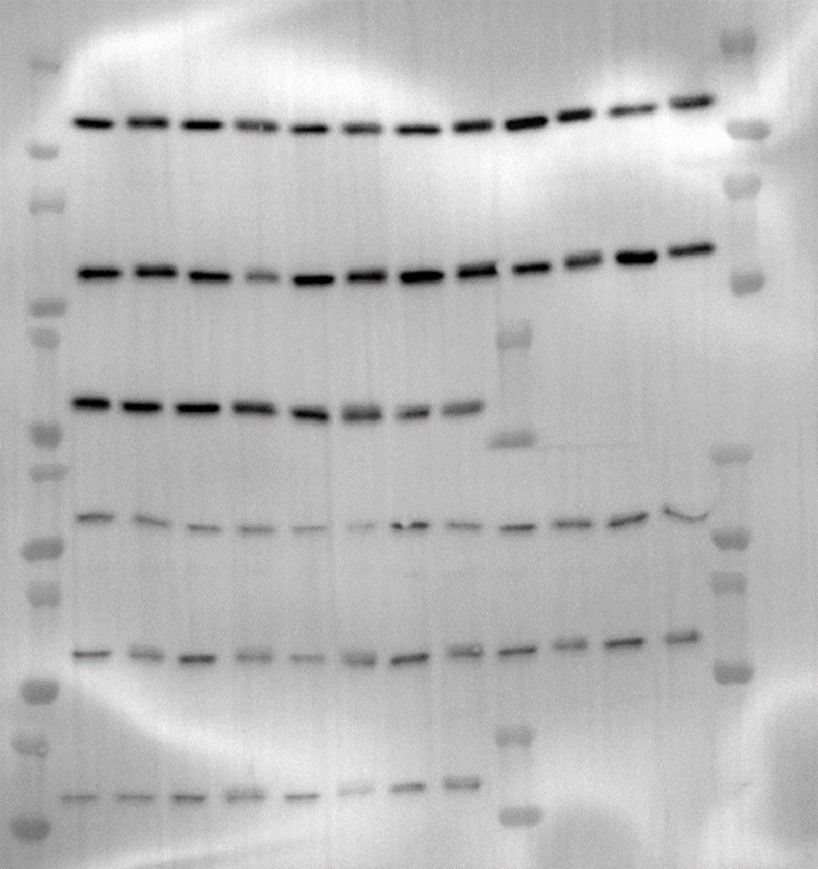

Supplement: Figure 3—figure supplement 1—source data 1. — Data used for quantification of Figure 3—figure supplement 1A, B, F, I and raw unedited blots for Figure 2—figure supplement 1A, B. [file elife-83338-fig3-figsupp1-data1.zip › Figure 3 - supplement 1/Figure 3 - figure supplement 1 A+B Akt2 membrane 2.jpg]

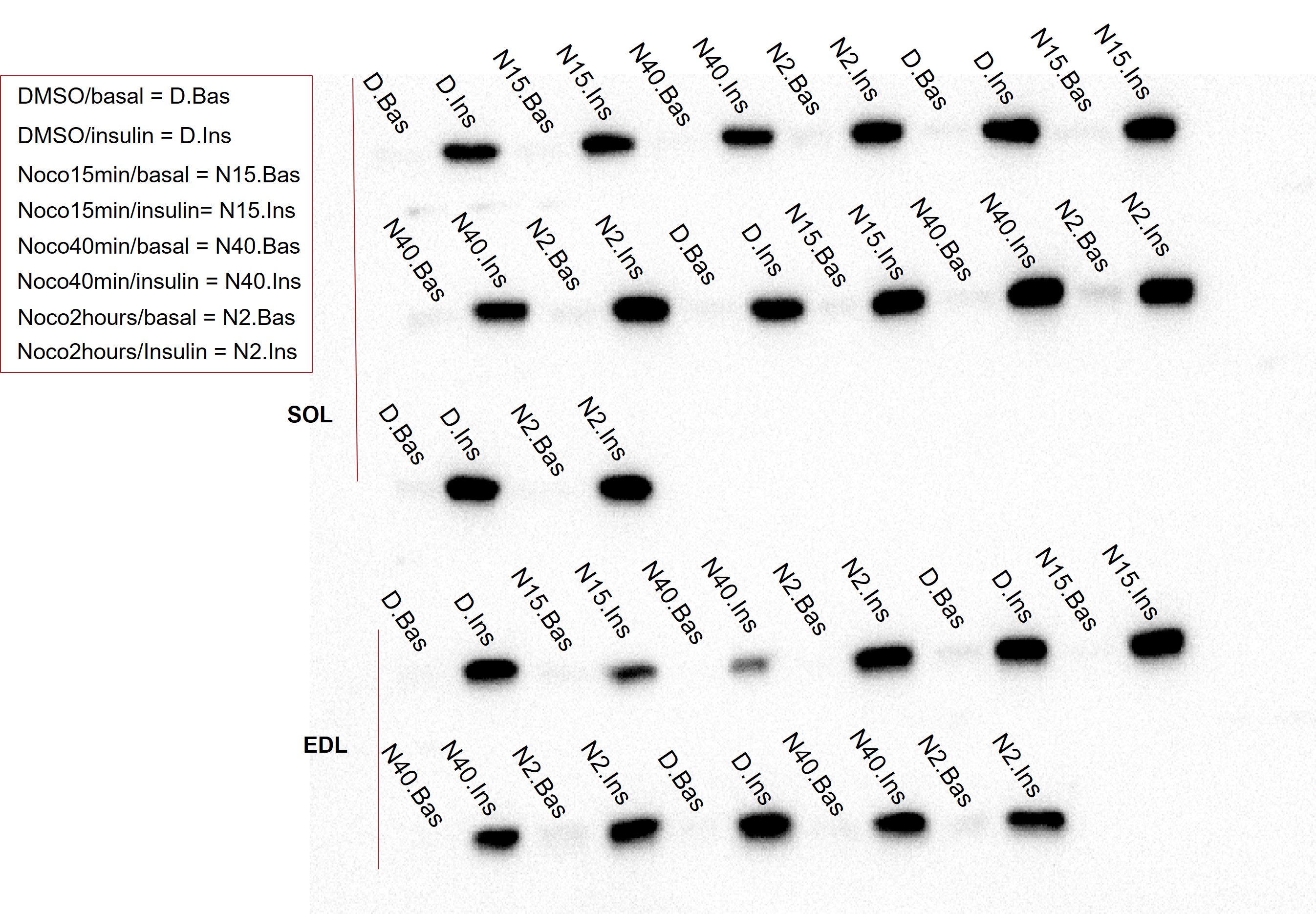

Supplement: Figure 3—figure supplement 1—source data 1. — Data used for quantification of Figure 3—figure supplement 1A, B, F, I and raw unedited blots for Figure 2—figure supplement 1A, B. [file elife-83338-fig3-figsupp1-data1.zip › Figure 3 - supplement 1/Figure 3 - figure supplement 1 A+B p-Akt 308 membrane 1 marked.jpg]

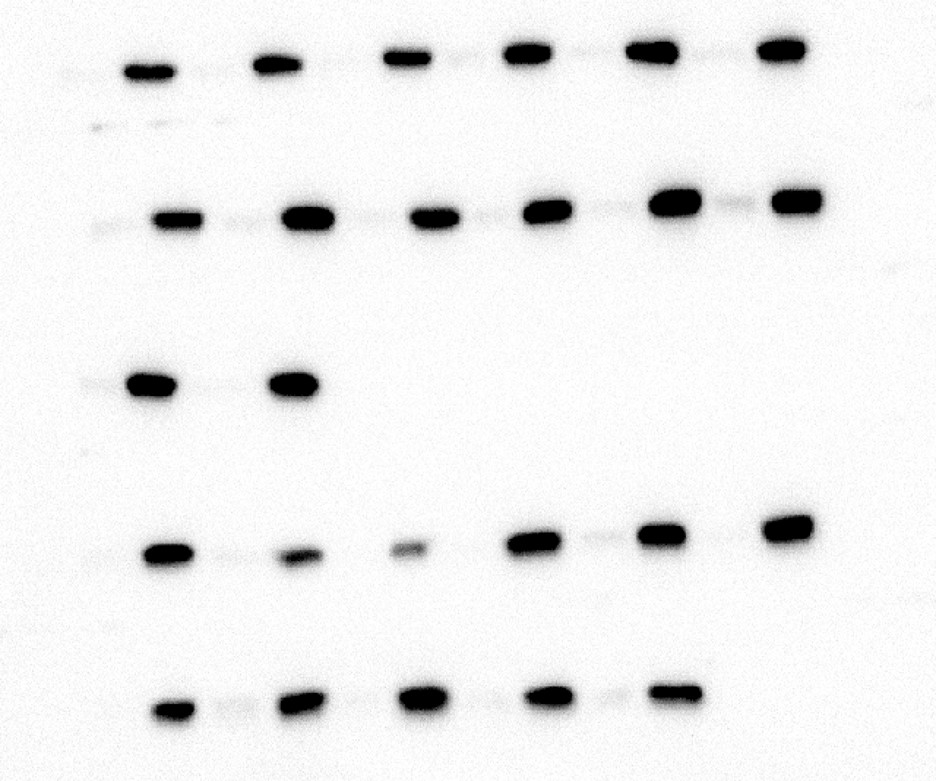

Supplement: Figure 3—figure supplement 1—source data 1. — Data used for quantification of Figure 3—figure supplement 1A, B, F, I and raw unedited blots for Figure 2—figure supplement 1A, B. [file elife-83338-fig3-figsupp1-data1.zip › Figure 3 - supplement 1/Figure 3 - figure supplement 1 A+B p-Akt 308 membrane 1.jpg]

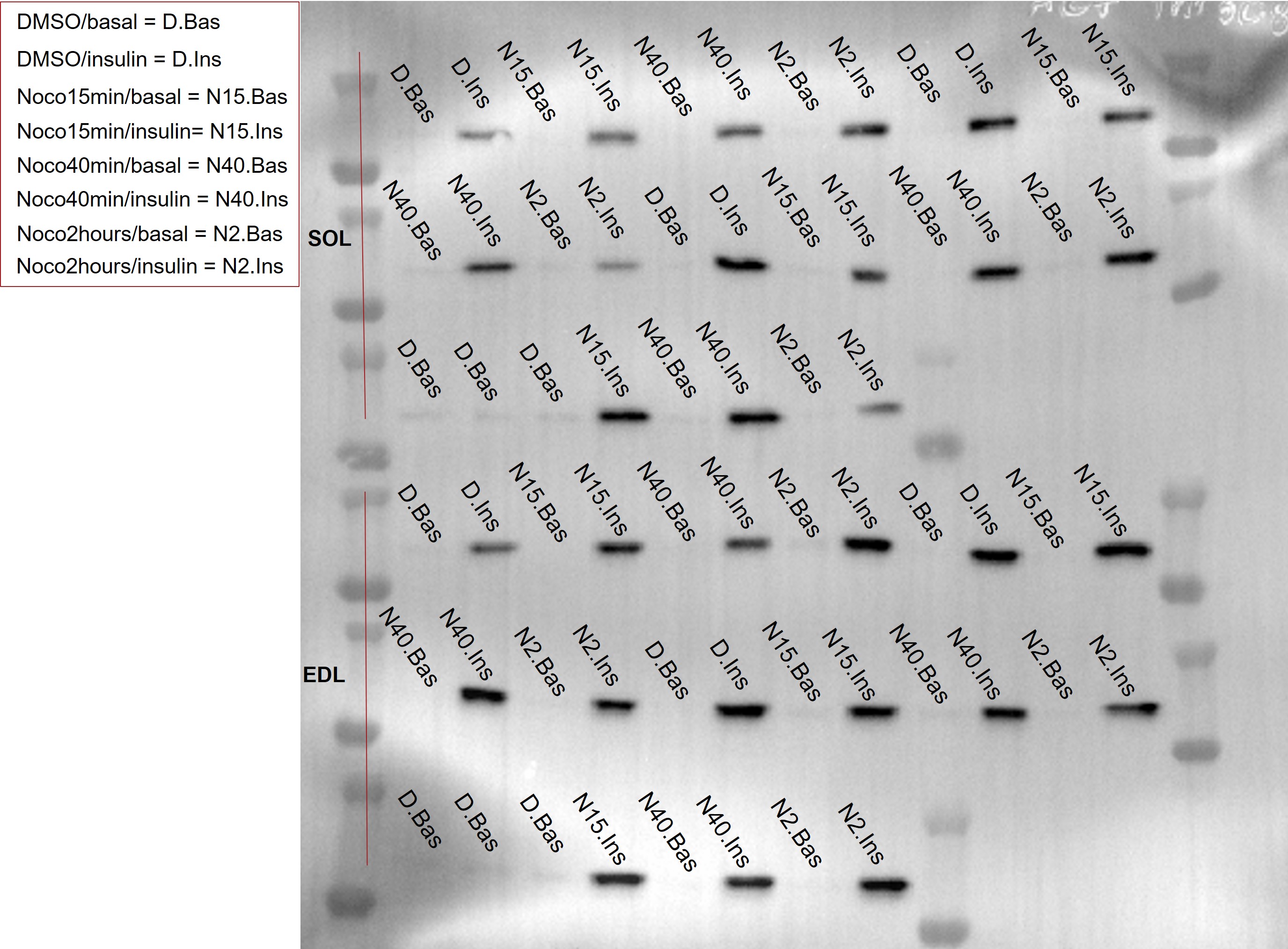

Supplement: Figure 3—figure supplement 1—source data 1. — Data used for quantification of Figure 3—figure supplement 1A, B, F, I and raw unedited blots for Figure 2—figure supplement 1A, B. [file elife-83338-fig3-figsupp1-data1.zip › Figure 3 - supplement 1/Figure 3 - figure supplement 1 A+B p-Akt 308 membrane 2 marked.jpg]

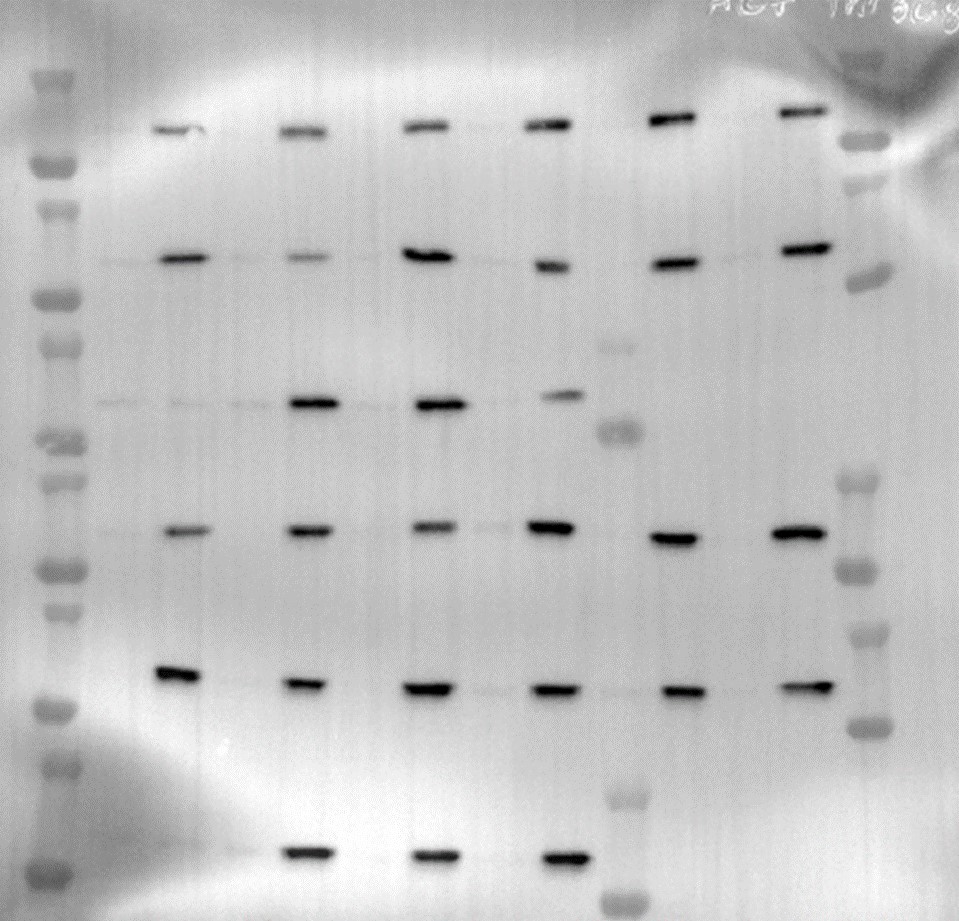

Supplement: Figure 3—figure supplement 1—source data 1. — Data used for quantification of Figure 3—figure supplement 1A, B, F, I and raw unedited blots for Figure 2—figure supplement 1A, B. [file elife-83338-fig3-figsupp1-data1.zip › Figure 3 - supplement 1/Figure 3 - figure supplement 1 A+B p-Akt 308 membrane 2.jpg]

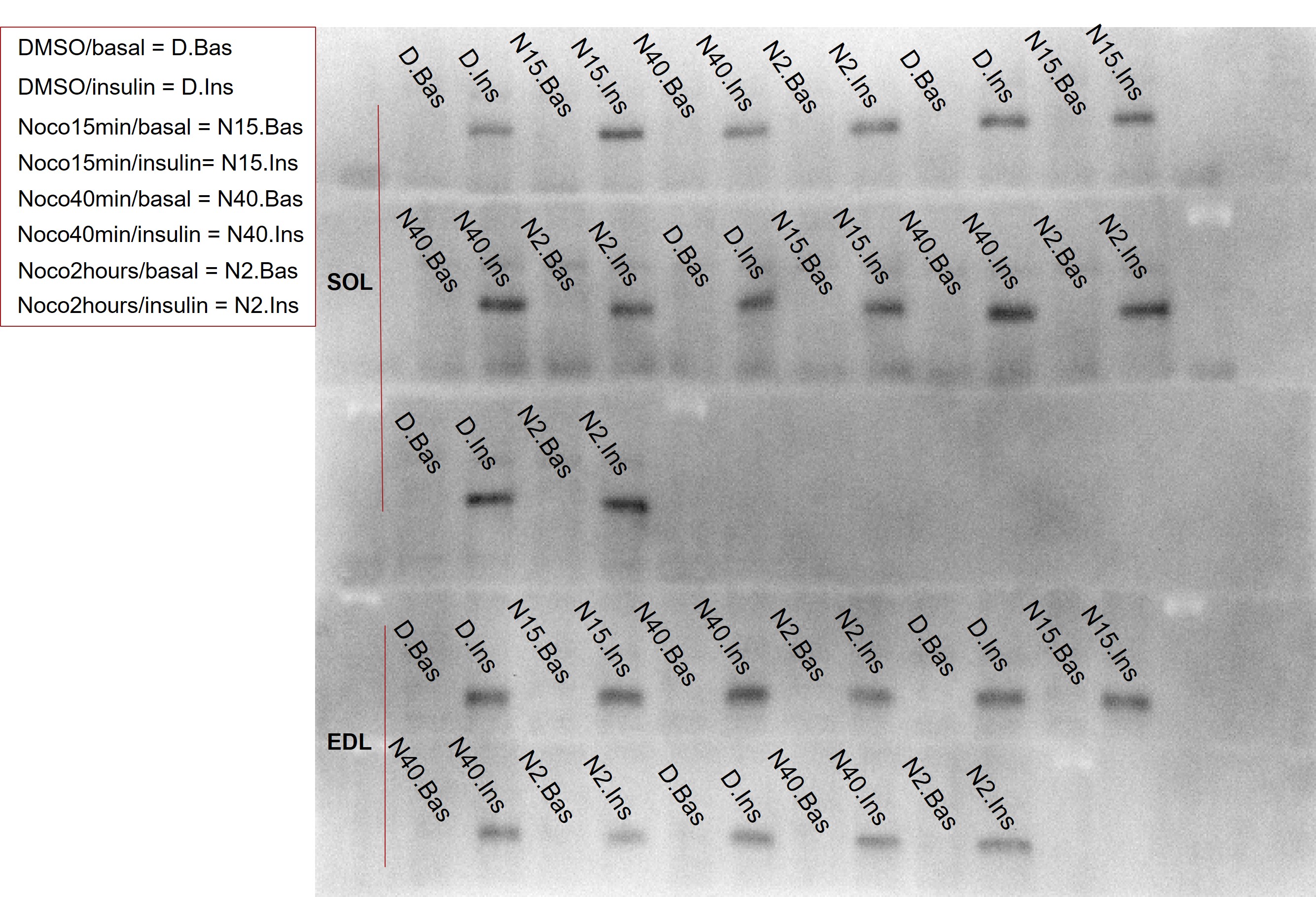

Supplement: Figure 3—figure supplement 1—source data 1. — Data used for quantification of Figure 3—figure supplement 1A, B, F, I and raw unedited blots for Figure 2—figure supplement 1A, B. [file elife-83338-fig3-figsupp1-data1.zip › Figure 3 - supplement 1/Figure 3 - figure supplement 1 A+B p-Akt 473 membrane 1 marked.jpg]

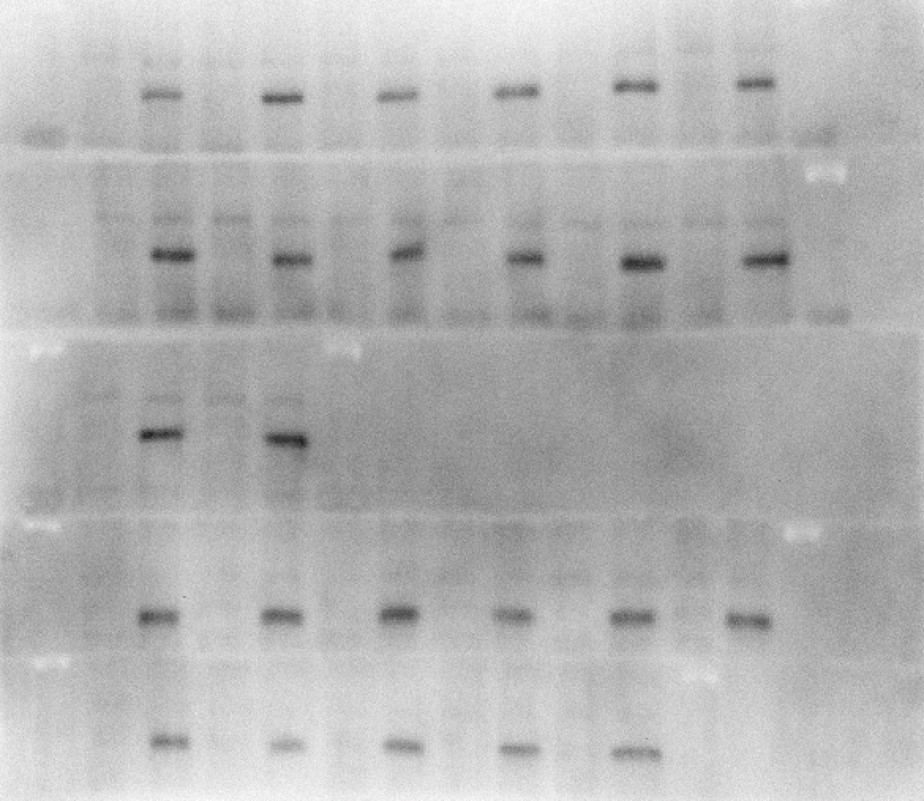

Supplement: Figure 3—figure supplement 1—source data 1. — Data used for quantification of Figure 3—figure supplement 1A, B, F, I and raw unedited blots for Figure 2—figure supplement 1A, B. [file elife-83338-fig3-figsupp1-data1.zip › Figure 3 - supplement 1/Figure 3 - figure supplement 1 A+B p-Akt 473 membrane 1.jpg]

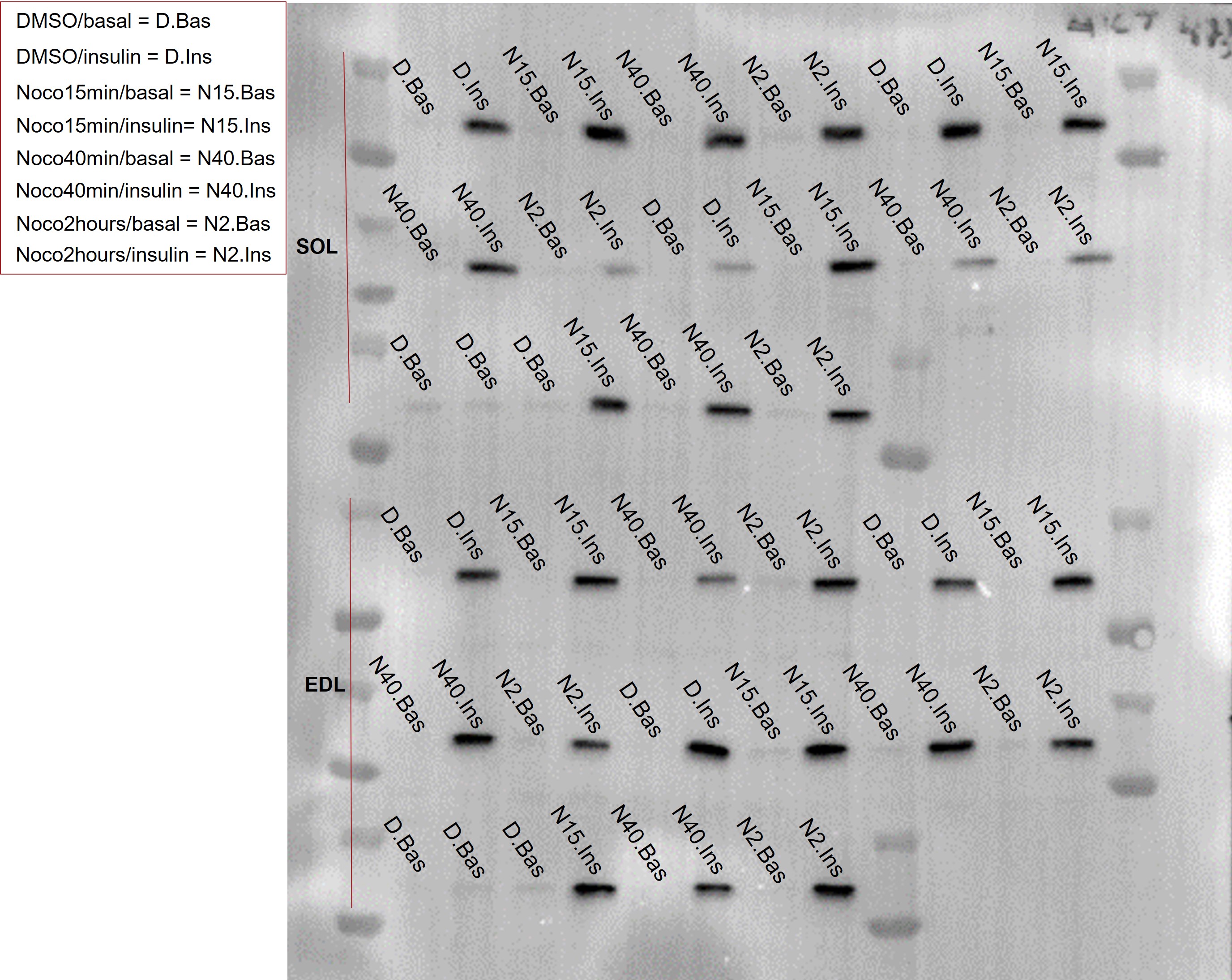

Supplement: Figure 3—figure supplement 1—source data 1. — Data used for quantification of Figure 3—figure supplement 1A, B, F, I and raw unedited blots for Figure 2—figure supplement 1A, B. [file elife-83338-fig3-figsupp1-data1.zip › Figure 3 - supplement 1/Figure 3 - figure supplement 1 A+B p-Akt 473 membrane 2 marked.jpg]

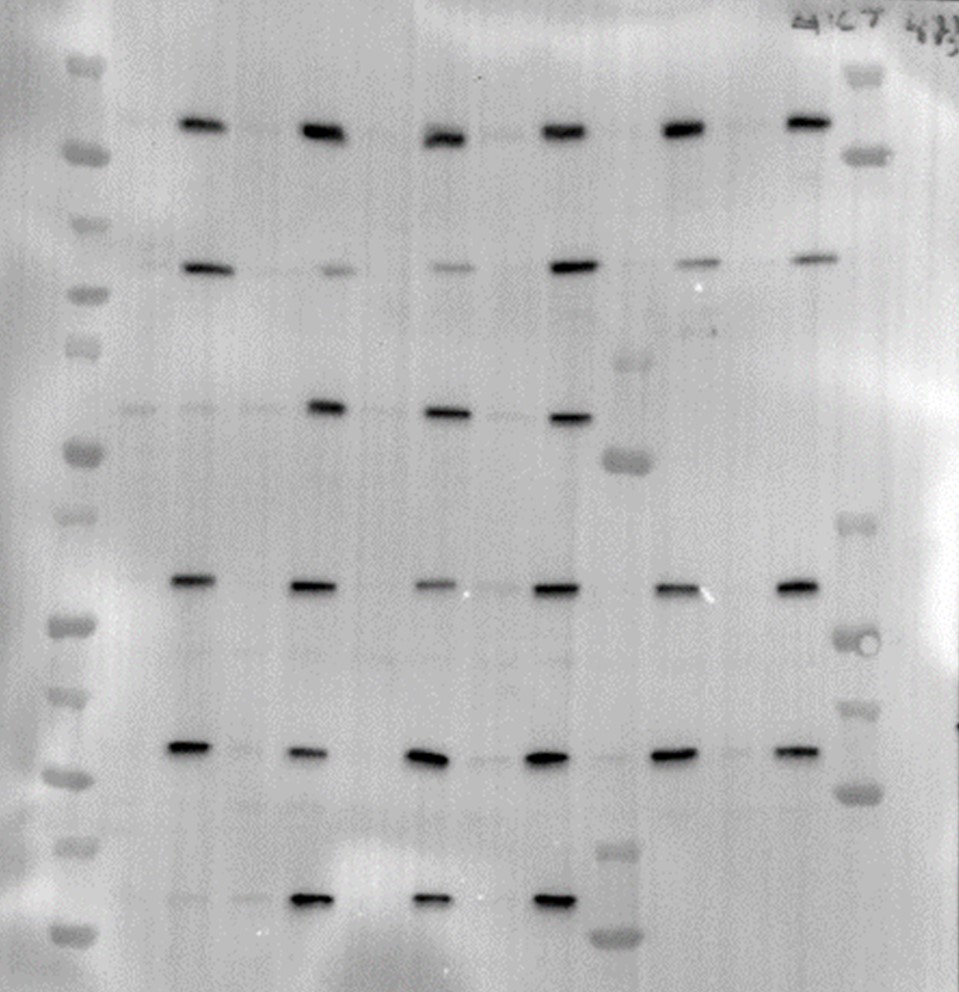

Supplement: Figure 3—figure supplement 1—source data 1. — Data used for quantification of Figure 3—figure supplement 1A, B, F, I and raw unedited blots for Figure 2—figure supplement 1A, B. [file elife-83338-fig3-figsupp1-data1.zip › Figure 3 - supplement 1/Figure 3 - figure supplement 1 A+B p-Akt 473 membrane 2.jpg]

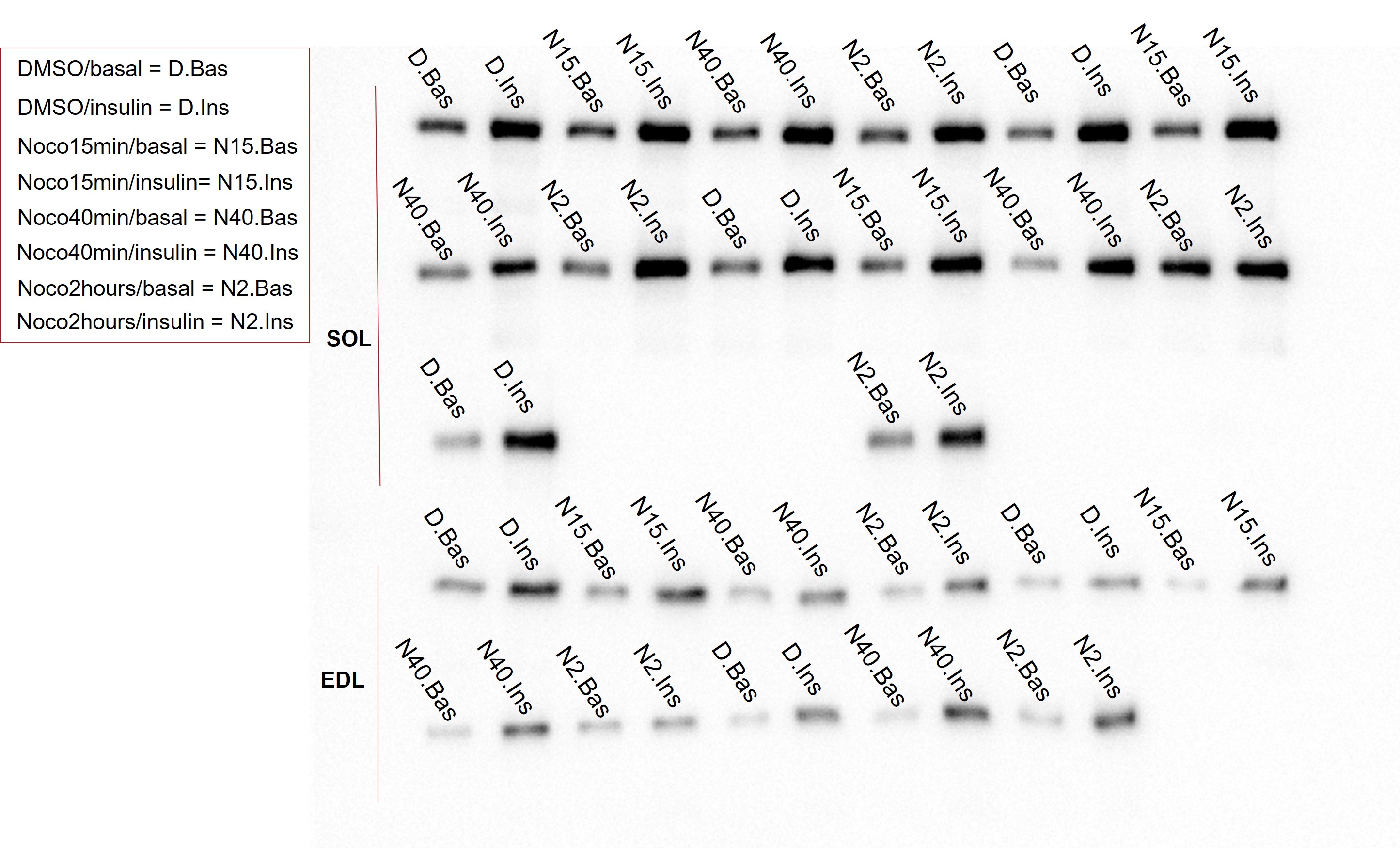

Supplement: Figure 3—figure supplement 1—source data 1. — Data used for quantification of Figure 3—figure supplement 1A, B, F, I and raw unedited blots for Figure 2—figure supplement 1A, B. [file elife-83338-fig3-figsupp1-data1.zip › Figure 3 - supplement 1/Figure 3 - figure supplement 1 A+B p-TBC1D4 595 membrane 1 marked.jpg]

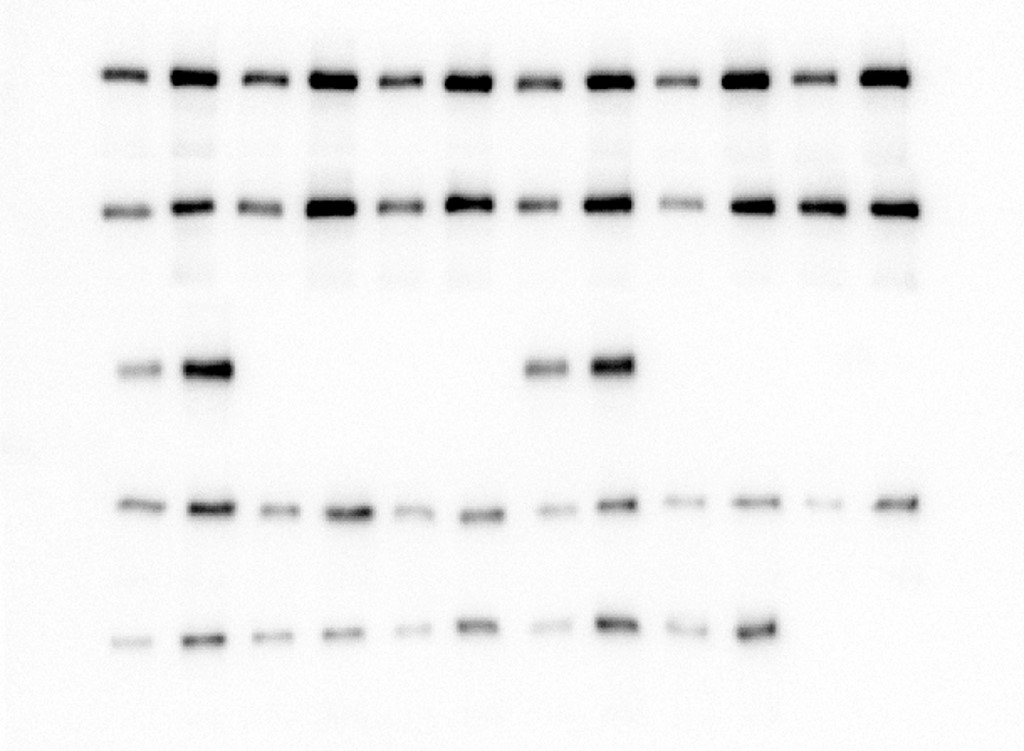

Supplement: Figure 3—figure supplement 1—source data 1. — Data used for quantification of Figure 3—figure supplement 1A, B, F, I and raw unedited blots for Figure 2—figure supplement 1A, B. [file elife-83338-fig3-figsupp1-data1.zip › Figure 3 - supplement 1/Figure 3 - figure supplement 1 A+B p-TBC1D4 595 membrane 1.jpg]

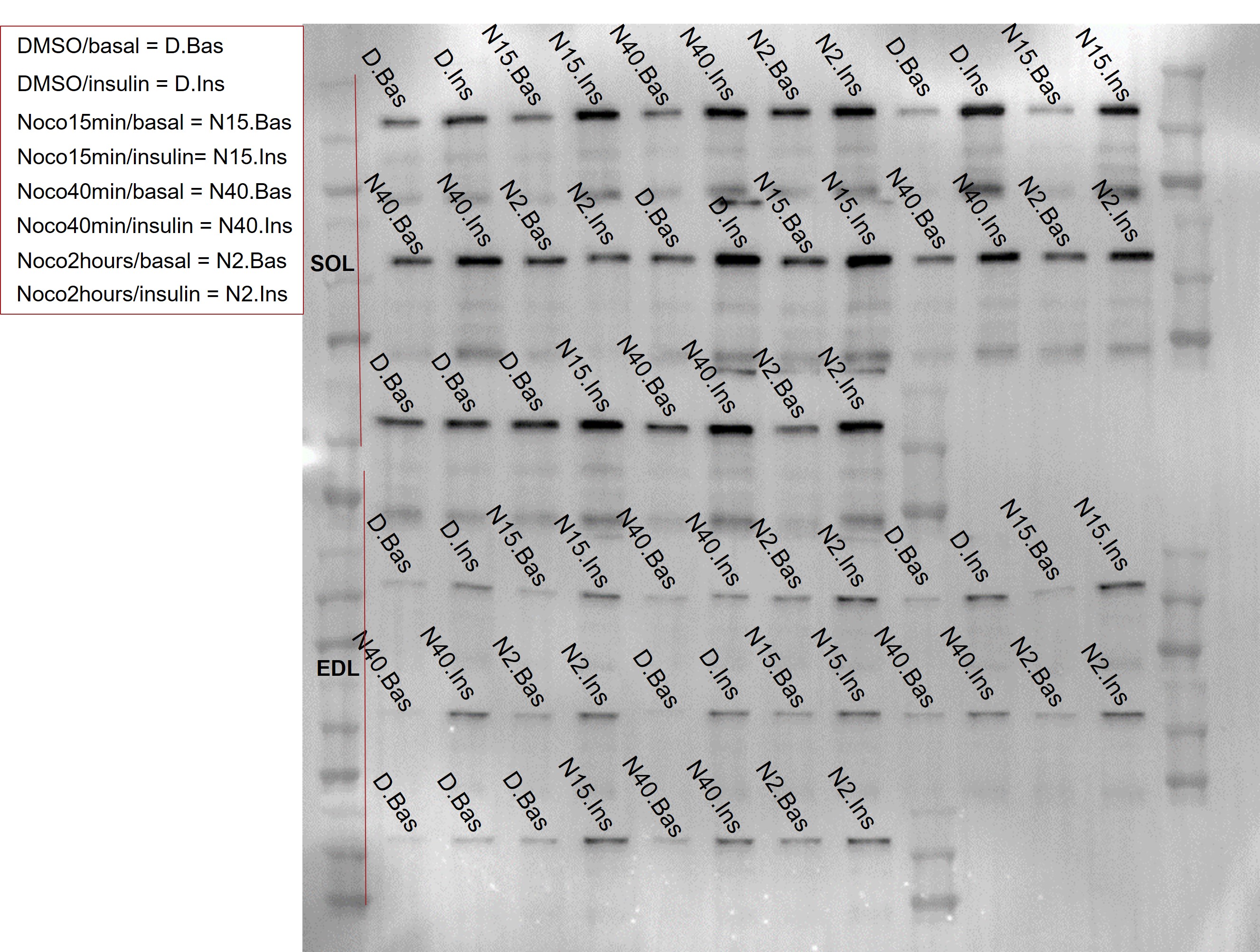

Supplement: Figure 3—figure supplement 1—source data 1. — Data used for quantification of Figure 3—figure supplement 1A, B, F, I and raw unedited blots for Figure 2—figure supplement 1A, B. [file elife-83338-fig3-figsupp1-data1.zip › Figure 3 - supplement 1/Figure 3 - figure supplement 1 A+B p-TBC1D4 595 membrane 2 marked.jpg]

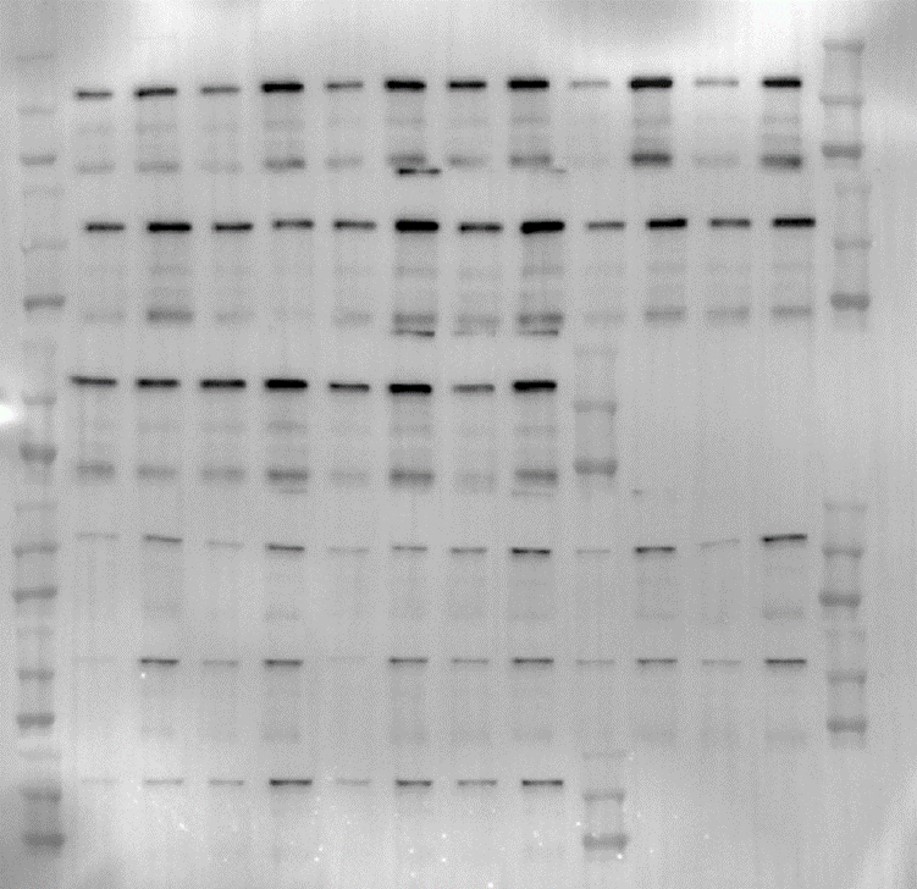

Supplement: Figure 3—figure supplement 1—source data 1. — Data used for quantification of Figure 3—figure supplement 1A, B, F, I and raw unedited blots for Figure 2—figure supplement 1A, B. [file elife-83338-fig3-figsupp1-data1.zip › Figure 3 - supplement 1/Figure 3 - figure supplement 1 A+B p-TBC1D4 595 membrane 2.jpg]

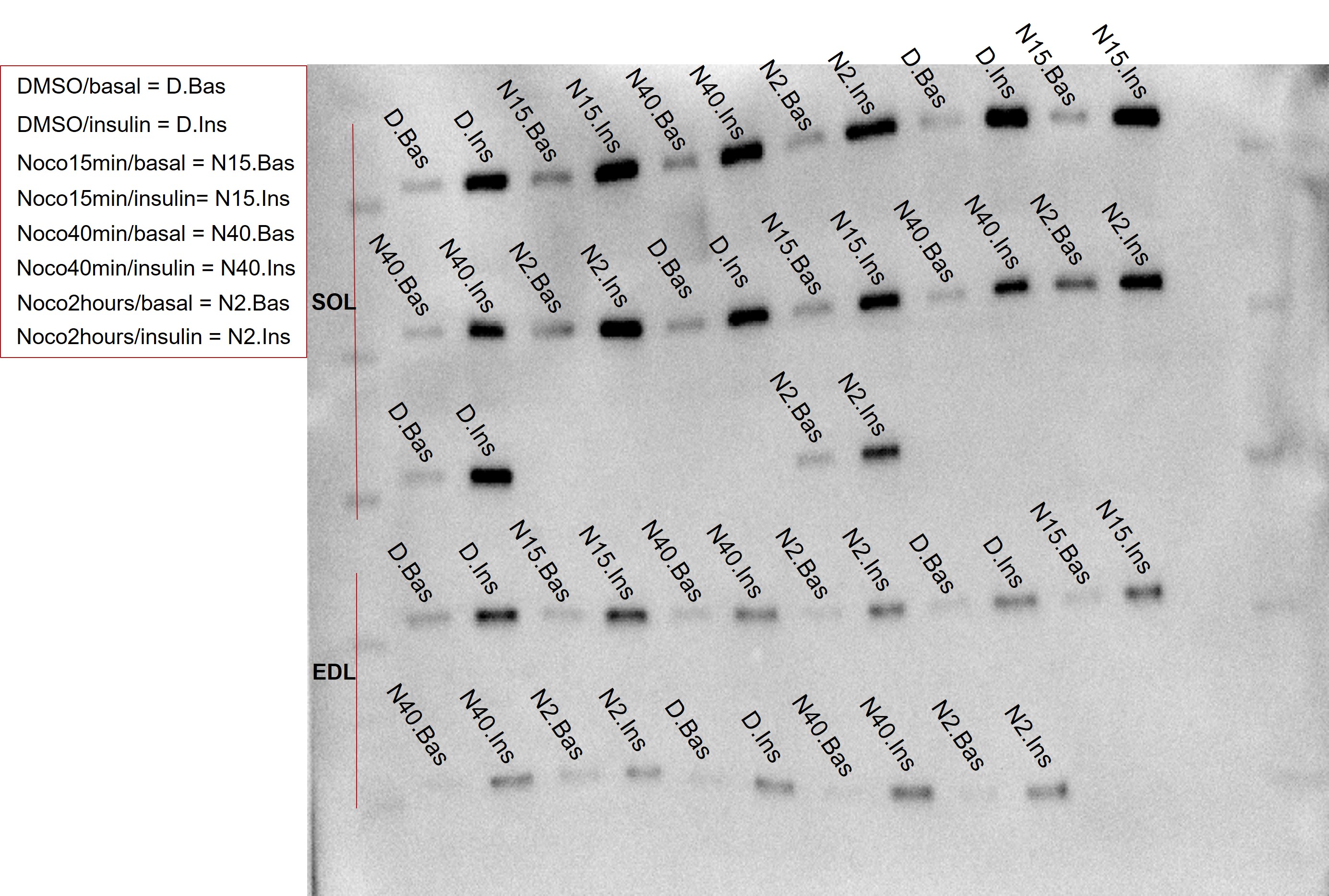

Supplement: Figure 3—figure supplement 1—source data 1. — Data used for quantification of Figure 3—figure supplement 1A, B, F, I and raw unedited blots for Figure 2—figure supplement 1A, B. [file elife-83338-fig3-figsupp1-data1.zip › Figure 3 - supplement 1/Figure 3 - figure supplement 1 A+B p-TBC1D4 649 membrane 1 marked.jpg]

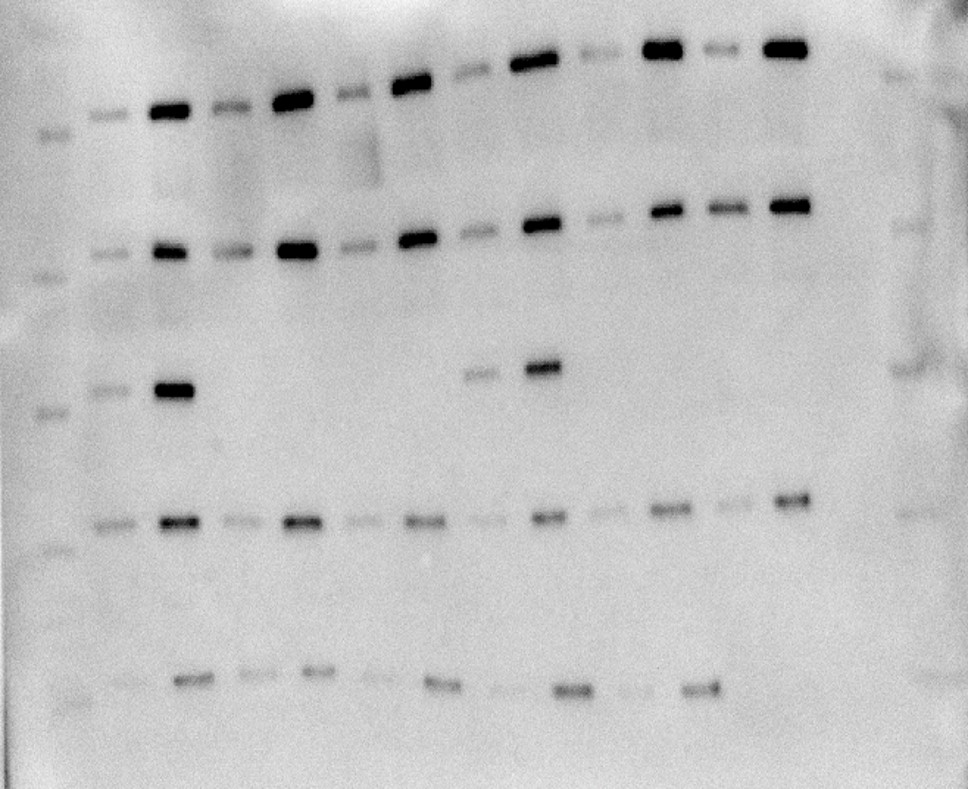

Supplement: Figure 3—figure supplement 1—source data 1. — Data used for quantification of Figure 3—figure supplement 1A, B, F, I and raw unedited blots for Figure 2—figure supplement 1A, B. [file elife-83338-fig3-figsupp1-data1.zip › Figure 3 - supplement 1/Figure 3 - figure supplement 1 A+B p-TBC1D4 649 membrane 1.jpg]

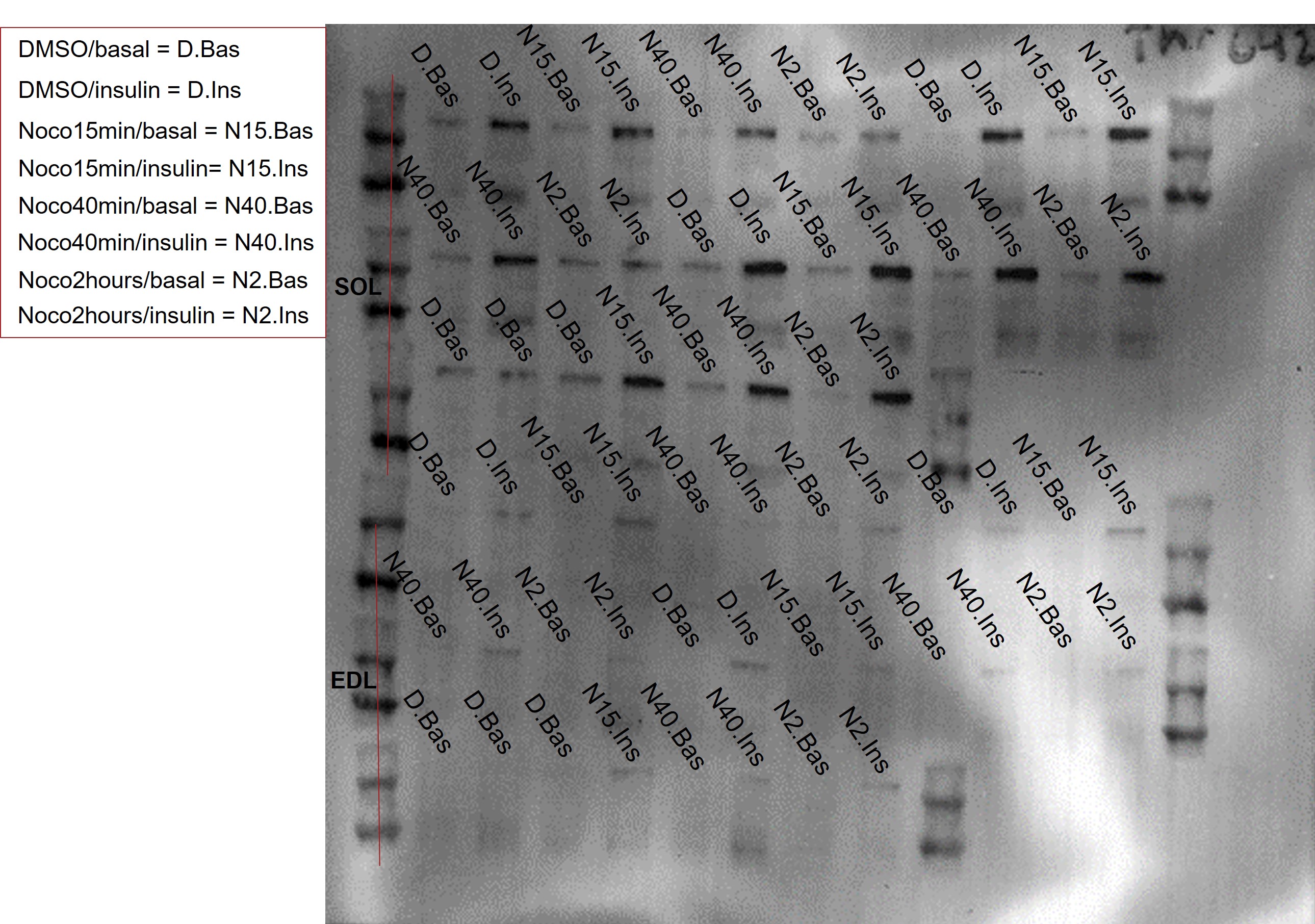

Supplement: Figure 3—figure supplement 1—source data 1. — Data used for quantification of Figure 3—figure supplement 1A, B, F, I and raw unedited blots for Figure 2—figure supplement 1A, B. [file elife-83338-fig3-figsupp1-data1.zip › Figure 3 - supplement 1/Figure 3 - figure supplement 1 A+B p-TBC1D4 649 membrane 2 marked.jpg]

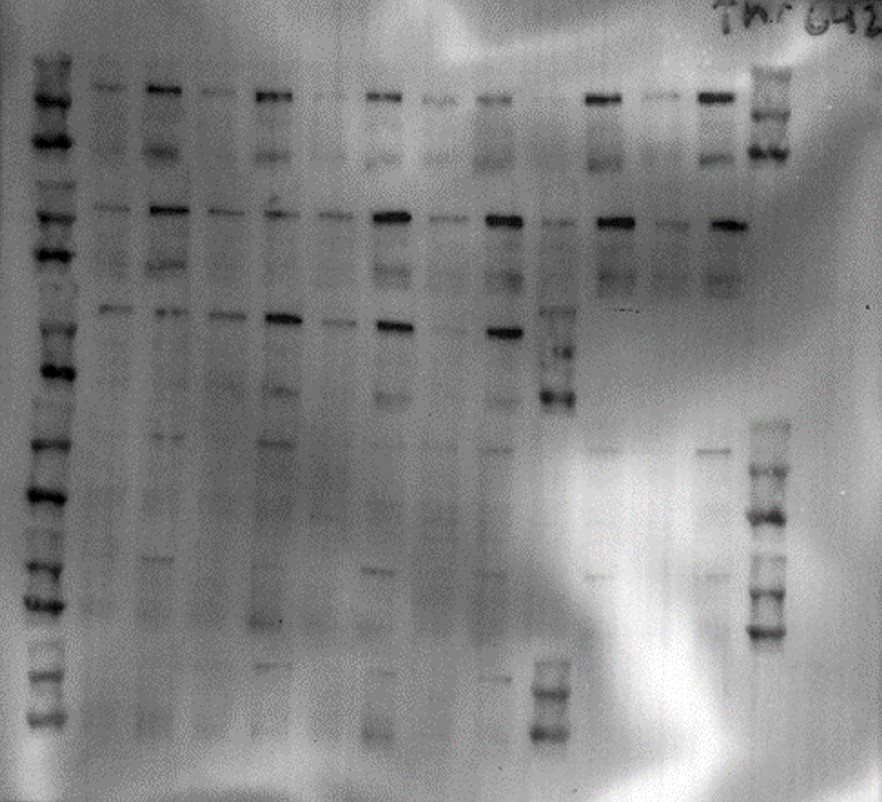

Supplement: Figure 3—figure supplement 1—source data 1. — Data used for quantification of Figure 3—figure supplement 1A, B, F, I and raw unedited blots for Figure 2—figure supplement 1A, B. [file elife-83338-fig3-figsupp1-data1.zip › Figure 3 - supplement 1/Figure 3 - figure supplement 1 A+B p-TBC1D4 649 membrane 2.jpg]

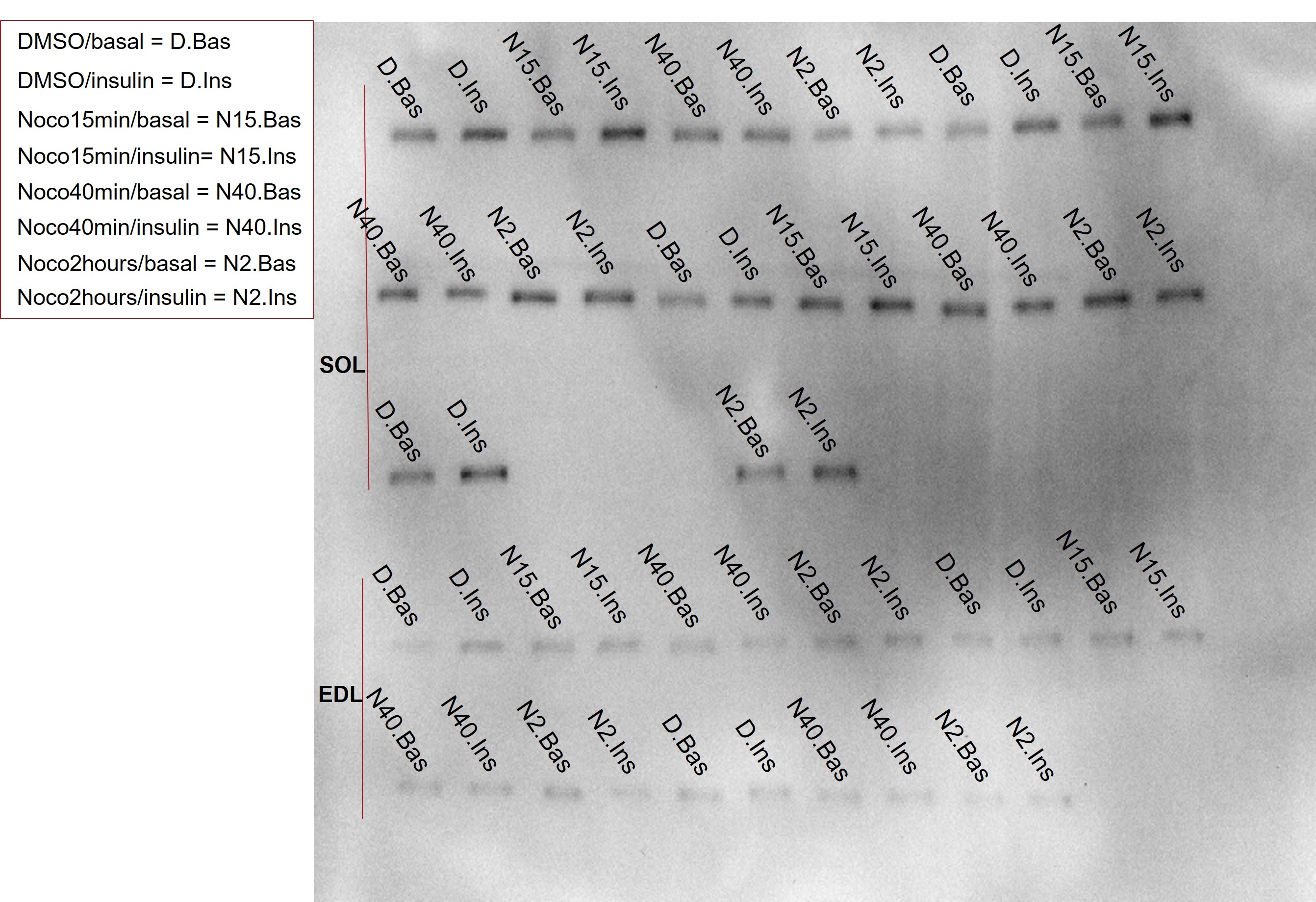

Supplement: Figure 3—figure supplement 1—source data 1. — Data used for quantification of Figure 3—figure supplement 1A, B, F, I and raw unedited blots for Figure 2—figure supplement 1A, B. [file elife-83338-fig3-figsupp1-data1.zip › Figure 3 - supplement 1/Figure 3 - figure supplement 1 A+B TBC1D4 membrane 1 marked.jpg]

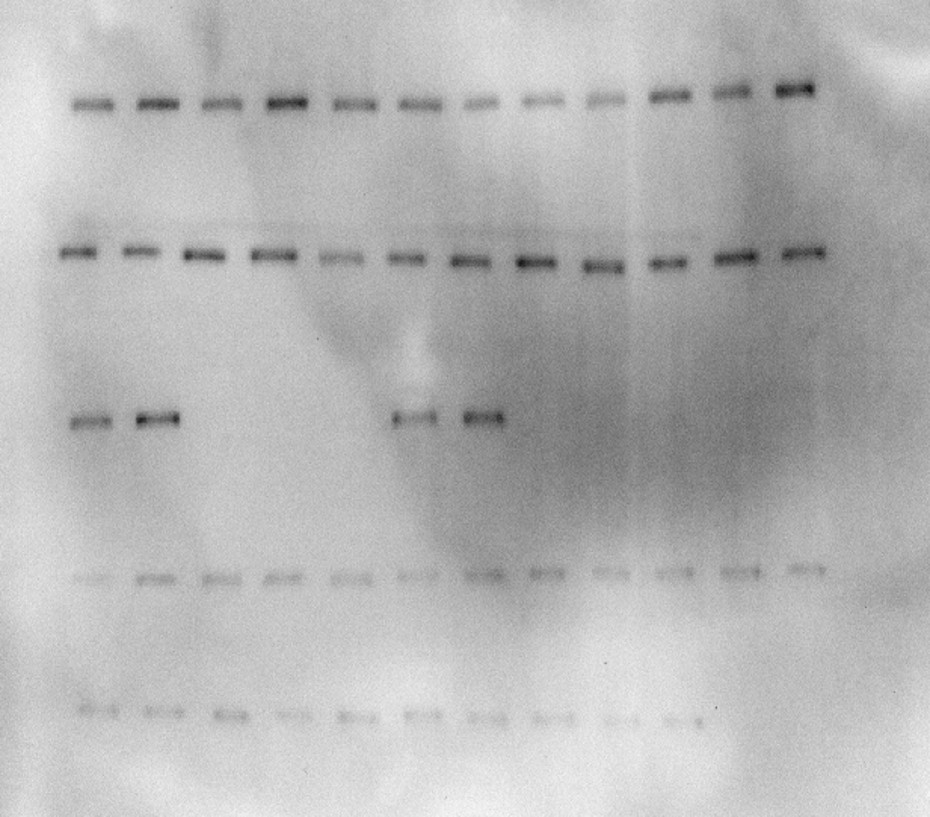

Supplement: Figure 3—figure supplement 1—source data 1. — Data used for quantification of Figure 3—figure supplement 1A, B, F, I and raw unedited blots for Figure 2—figure supplement 1A, B. [file elife-83338-fig3-figsupp1-data1.zip › Figure 3 - supplement 1/Figure 3 - figure supplement 1 A+B TBC1D4 membrane 1.jpg]

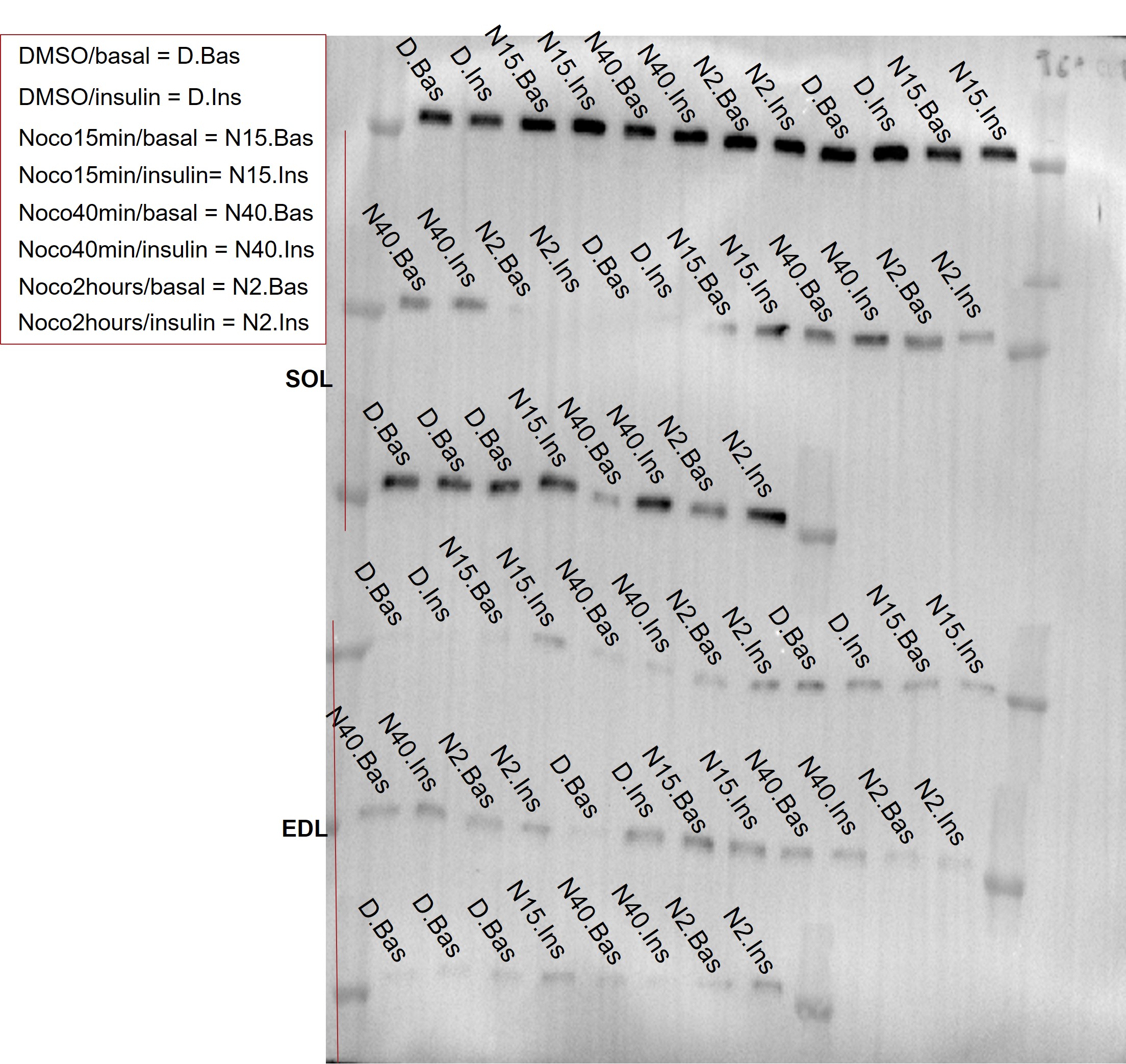

Supplement: Figure 3—figure supplement 1—source data 1. — Data used for quantification of Figure 3—figure supplement 1A, B, F, I and raw unedited blots for Figure 2—figure supplement 1A, B. [file elife-83338-fig3-figsupp1-data1.zip › Figure 3 - supplement 1/Figure 3 - figure supplement 1 A+B TBC1D4 membrane 2 marked.jpg]

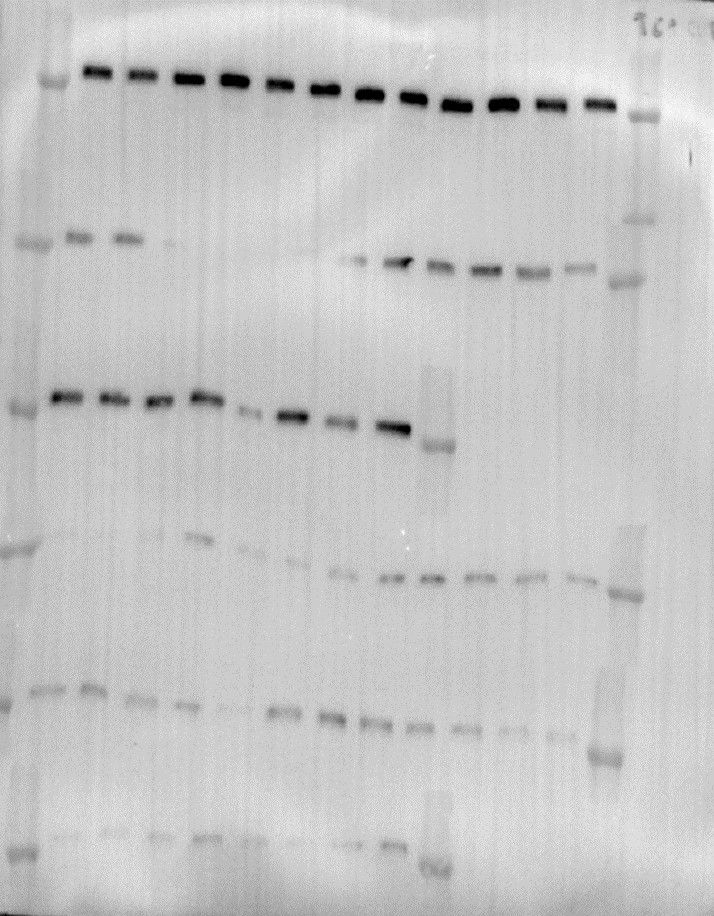

Supplement: Figure 3—figure supplement 1—source data 1. — Data used for quantification of Figure 3—figure supplement 1A, B, F, I and raw unedited blots for Figure 2—figure supplement 1A, B. [file elife-83338-fig3-figsupp1-data1.zip › Figure 3 - supplement 1/Figure 3 - figure supplement 1 A+B TBC1D4 membrane 2.jpg]

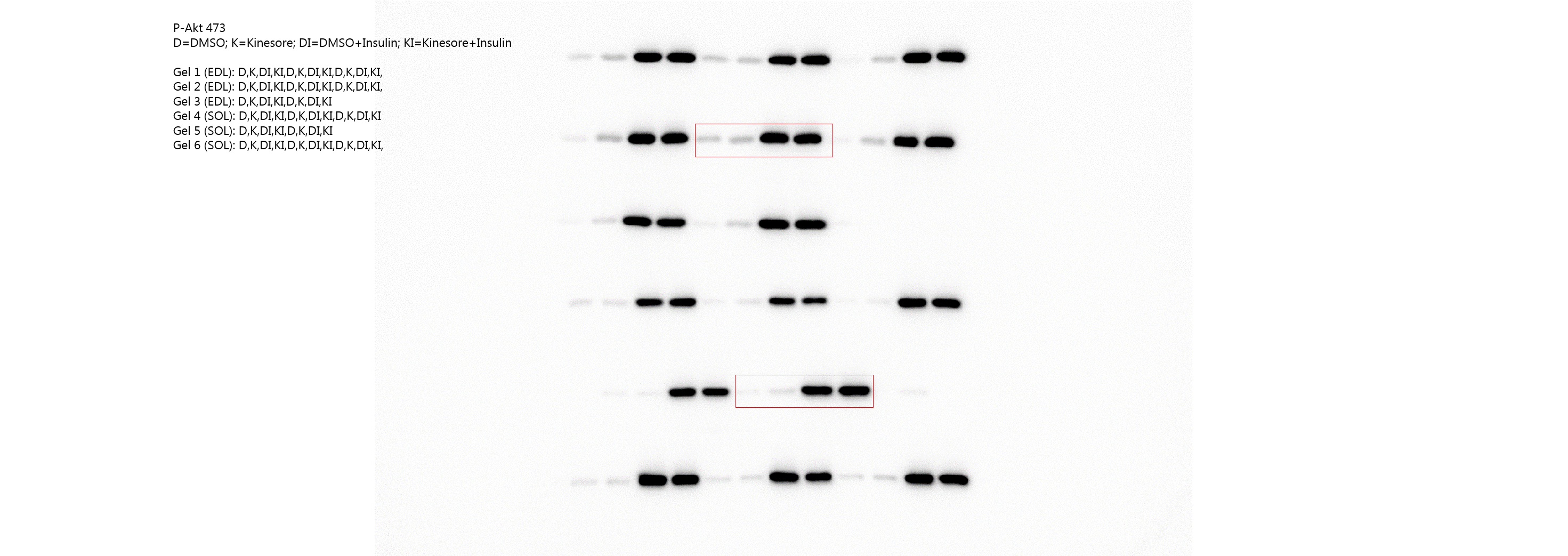

Supplement: Figure 4—figure supplement 1—source data 1. — Data used for quantification of Figure 4—figure supplement 1A–C, E–I and raw unedited blots for Figure 4—figure supplement 1A, B, D, I. [file elife-83338-fig4-figsupp1-data1.zip › Figure_4_-_supplement_1/Fig. 4 - supp. 1/Figure 4 - figure supplement 1 A AKT 473 EDL+SOL marked.png]

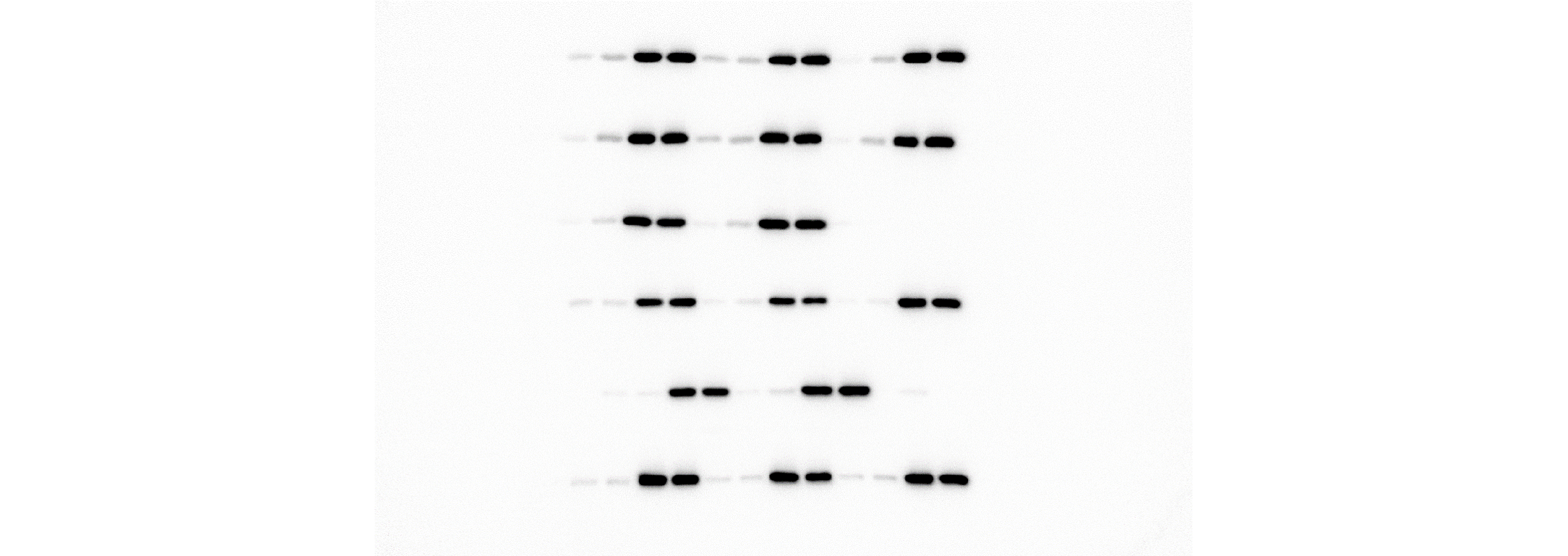

Supplement: Figure 4—figure supplement 1—source data 1. — Data used for quantification of Figure 4—figure supplement 1A–C, E–I and raw unedited blots for Figure 4—figure supplement 1A, B, D, I. [file elife-83338-fig4-figsupp1-data1.zip › Figure_4_-_supplement_1/Fig. 4 - supp. 1/Figure 4 - figure supplement 1 A AKT 473 EDL+SOL.png]

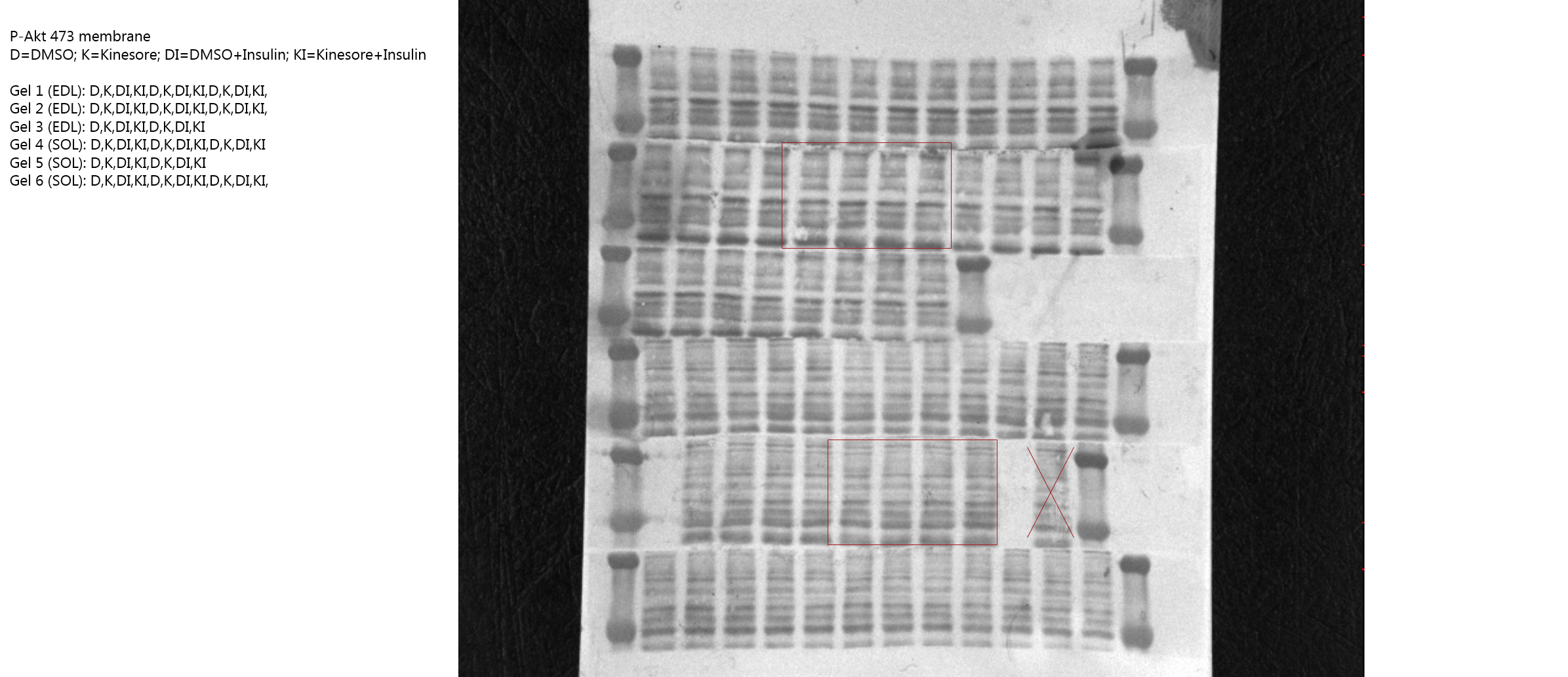

Supplement: Figure 4—figure supplement 1—source data 1. — Data used for quantification of Figure 4—figure supplement 1A–C, E–I and raw unedited blots for Figure 4—figure supplement 1A, B, D, I. [file elife-83338-fig4-figsupp1-data1.zip › Figure_4_-_supplement_1/Fig. 4 - supp. 1/Figure 4 - figure supplement 1 A Coomassie EDL+SOL marked.png]

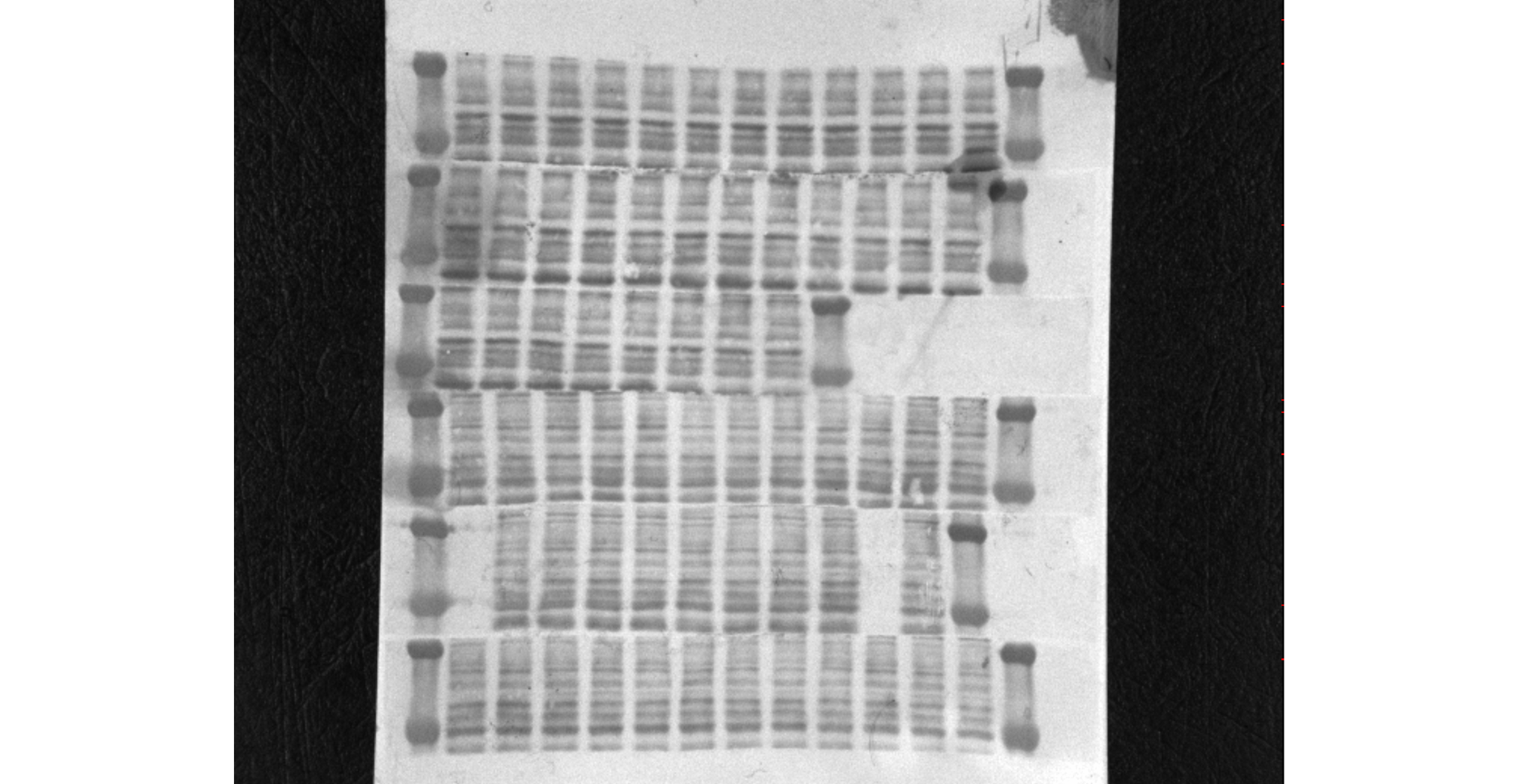

Supplement: Figure 4—figure supplement 1—source data 1. — Data used for quantification of Figure 4—figure supplement 1A–C, E–I and raw unedited blots for Figure 4—figure supplement 1A, B, D, I. [file elife-83338-fig4-figsupp1-data1.zip › Figure_4_-_supplement_1/Fig. 4 - supp. 1/Figure 4 - figure supplement 1 A Coomassie EDL+SOL.png]

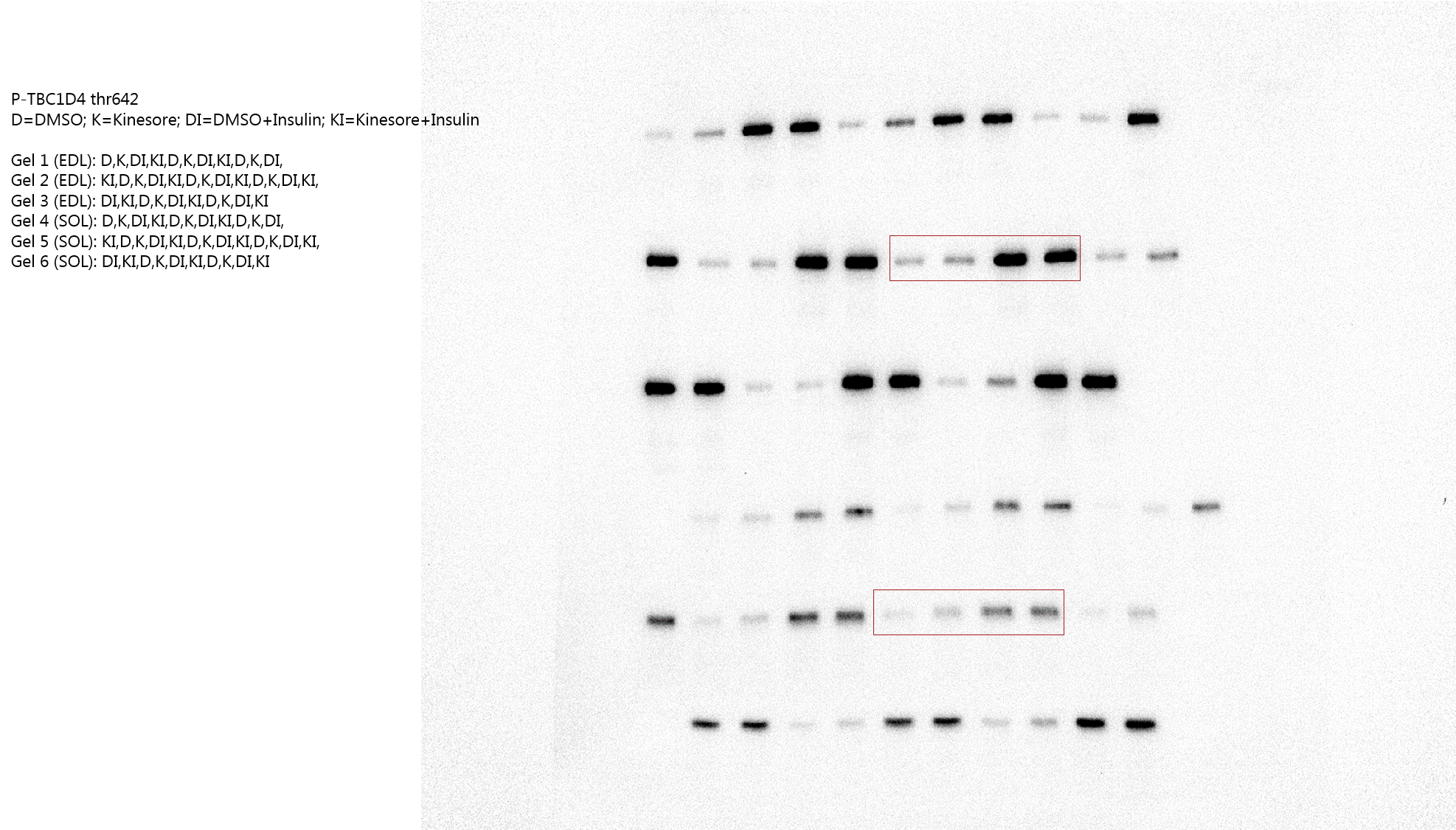

Supplement: Figure 4—figure supplement 1—source data 1. — Data used for quantification of Figure 4—figure supplement 1A–C, E–I and raw unedited blots for Figure 4—figure supplement 1A, B, D, I. [file elife-83338-fig4-figsupp1-data1.zip › Figure_4_-_supplement_1/Fig. 4 - supp. 1/Figure 4 - figure supplement 1 A TBC1D4 642 EDL+SOL marked.png]

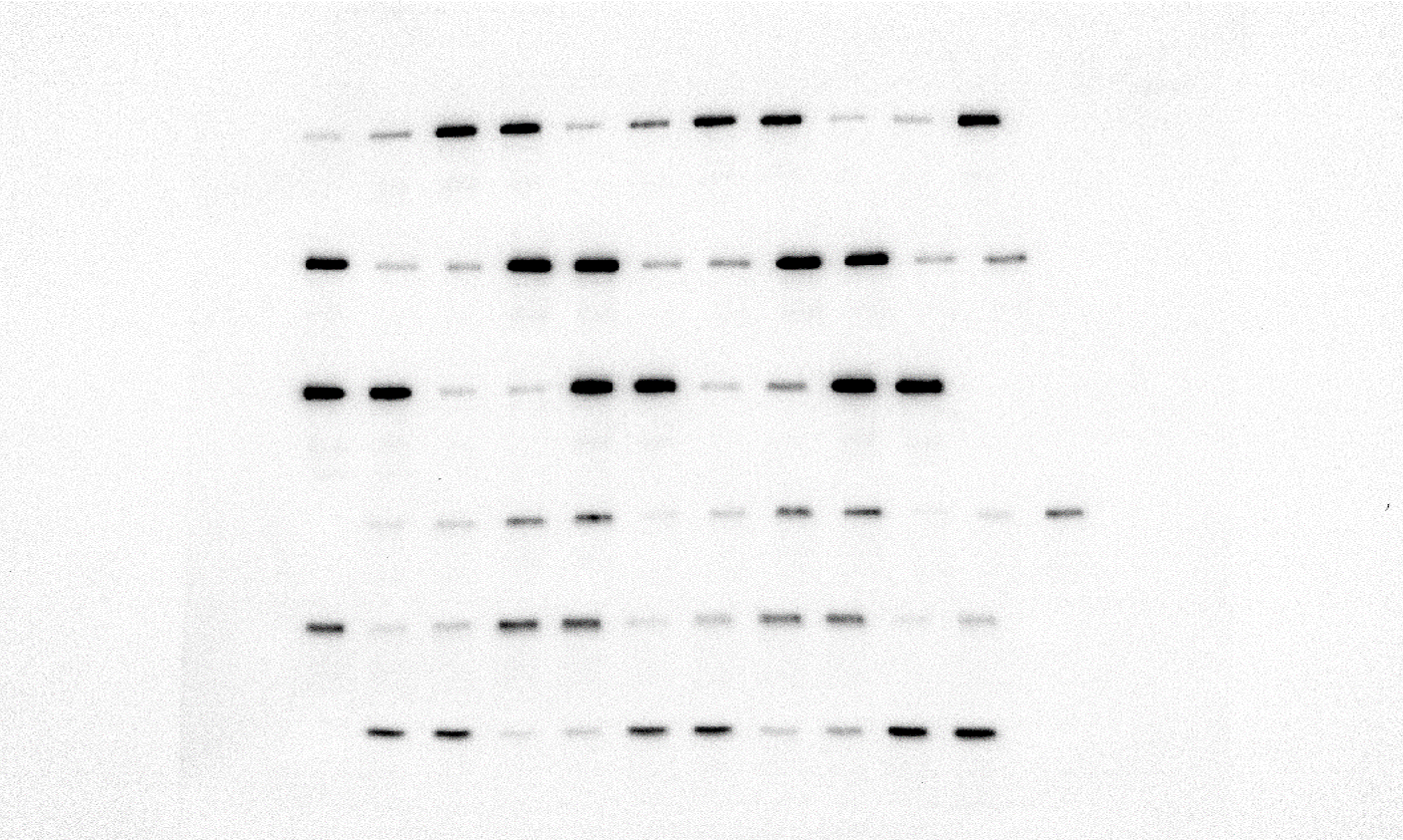

Supplement: Figure 4—figure supplement 1—source data 1. — Data used for quantification of Figure 4—figure supplement 1A–C, E–I and raw unedited blots for Figure 4—figure supplement 1A, B, D, I. [file elife-83338-fig4-figsupp1-data1.zip › Figure_4_-_supplement_1/Fig. 4 - supp. 1/Figure 4 - figure supplement 1 A TBC1D4 642 EDL+SOL.png]

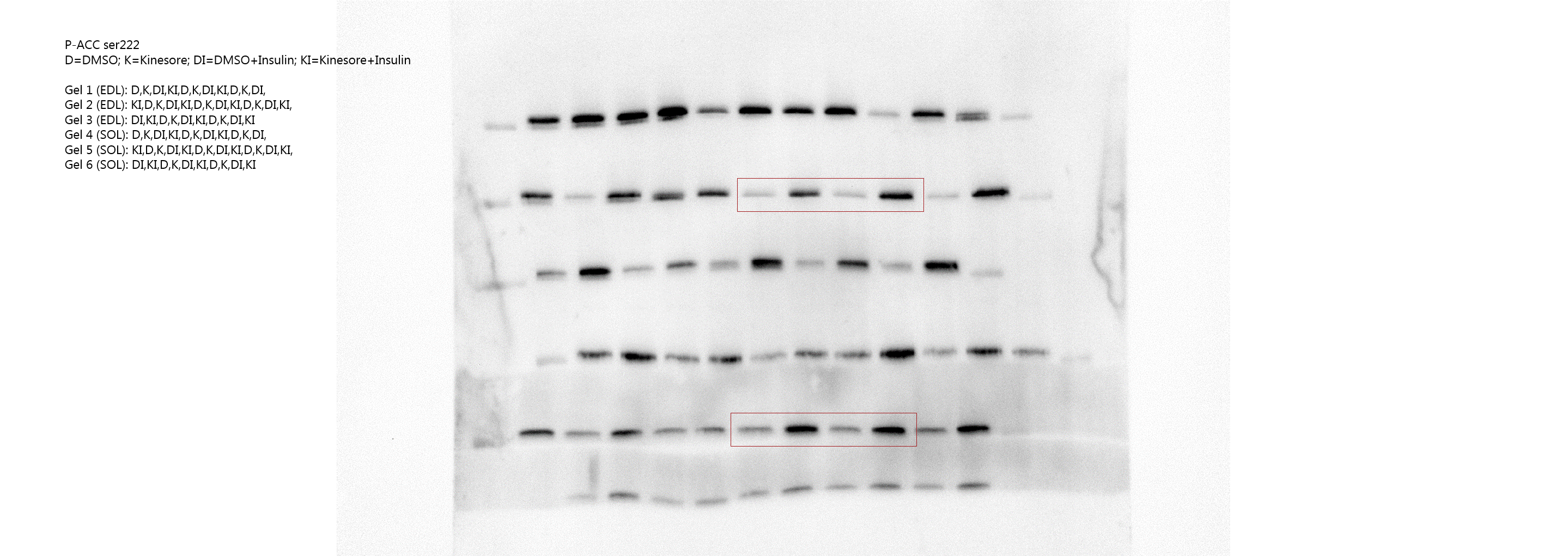

Supplement: Figure 4—figure supplement 1—source data 1. — Data used for quantification of Figure 4—figure supplement 1A–C, E–I and raw unedited blots for Figure 4—figure supplement 1A, B, D, I. [file elife-83338-fig4-figsupp1-data1.zip › Figure_4_-_supplement_1/Fig. 4 - supp. 1/Figure 4 - figure supplement 1 B ACC 222 EDL+SOL marked.png]

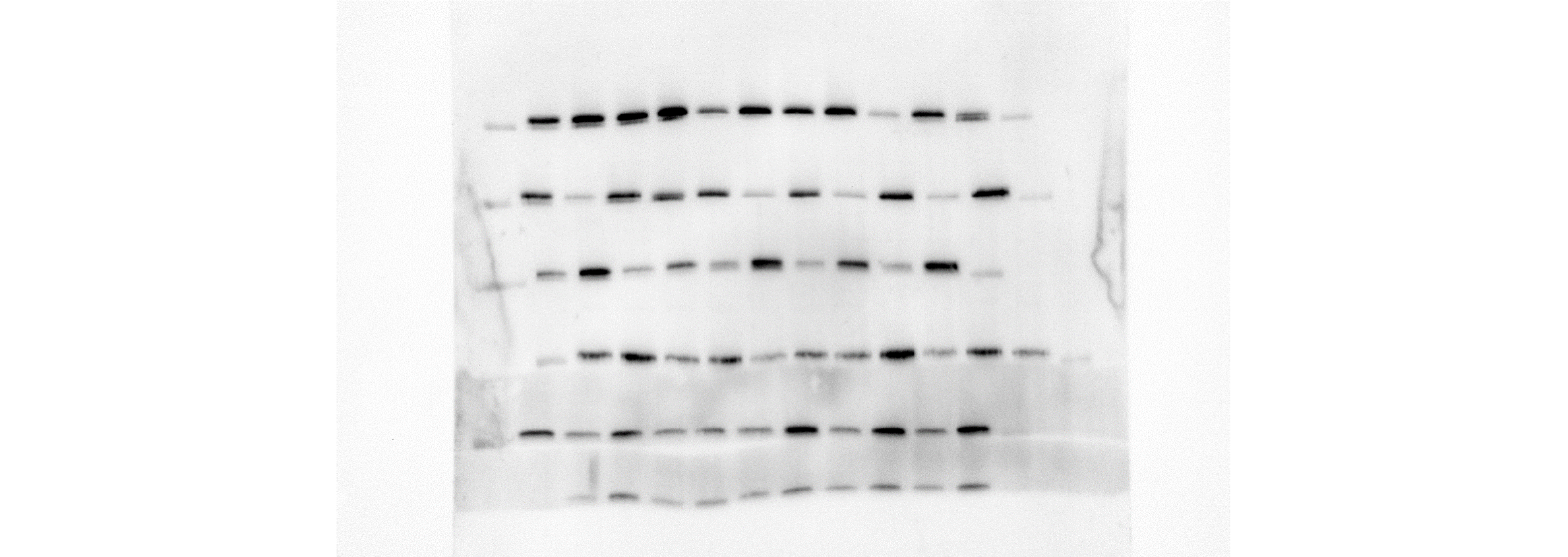

Supplement: Figure 4—figure supplement 1—source data 1. — Data used for quantification of Figure 4—figure supplement 1A–C, E–I and raw unedited blots for Figure 4—figure supplement 1A, B, D, I. [file elife-83338-fig4-figsupp1-data1.zip › Figure_4_-_supplement_1/Fig. 4 - supp. 1/Figure 4 - figure supplement 1 B ACC 222 EDL+SOL.png]

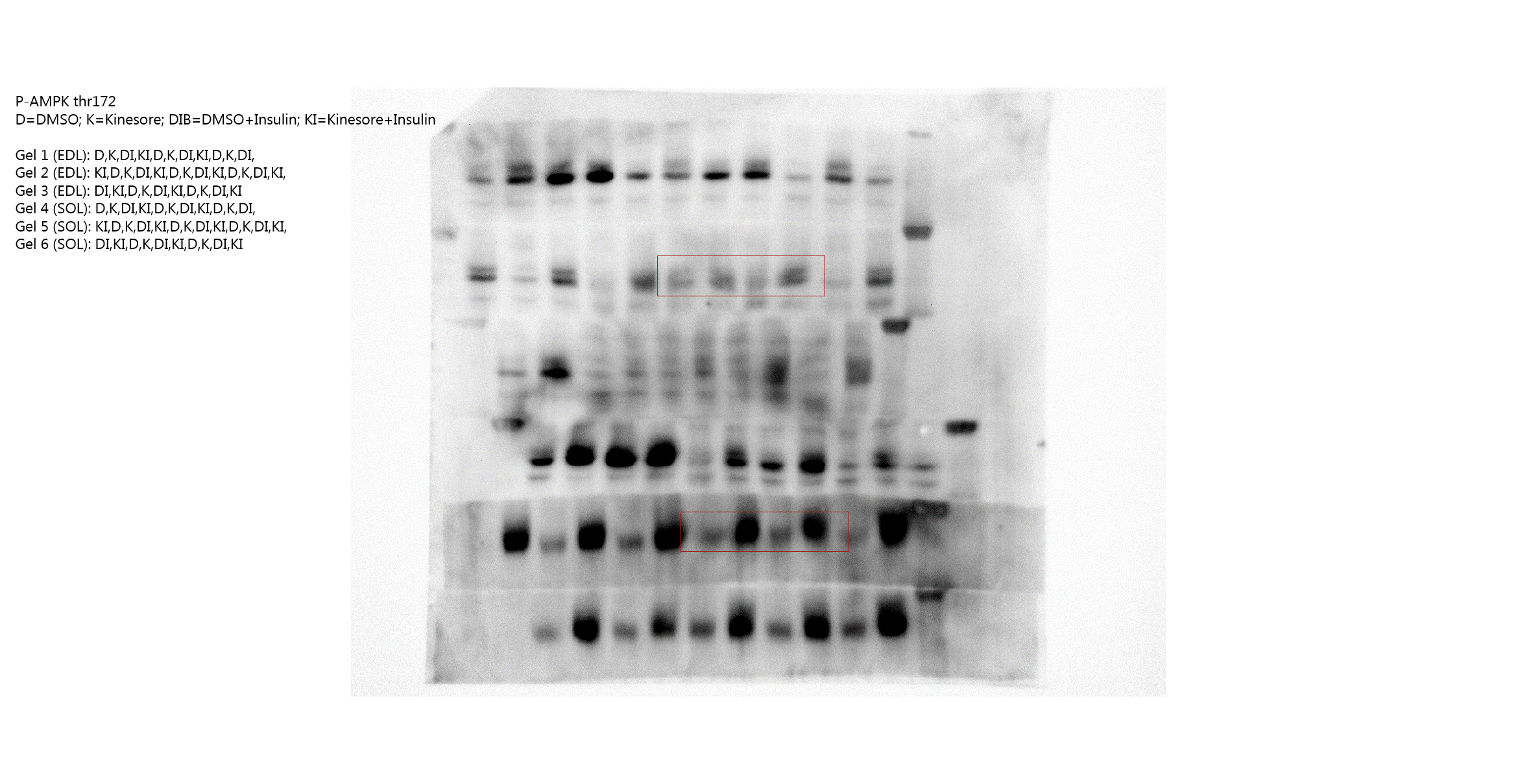

Supplement: Figure 4—figure supplement 1—source data 1. — Data used for quantification of Figure 4—figure supplement 1A–C, E–I and raw unedited blots for Figure 4—figure supplement 1A, B, D, I. [file elife-83338-fig4-figsupp1-data1.zip › Figure_4_-_supplement_1/Fig. 4 - supp. 1/Figure 4 - figure supplement 1 B AMPK 172 EDL+SOL marked.png]

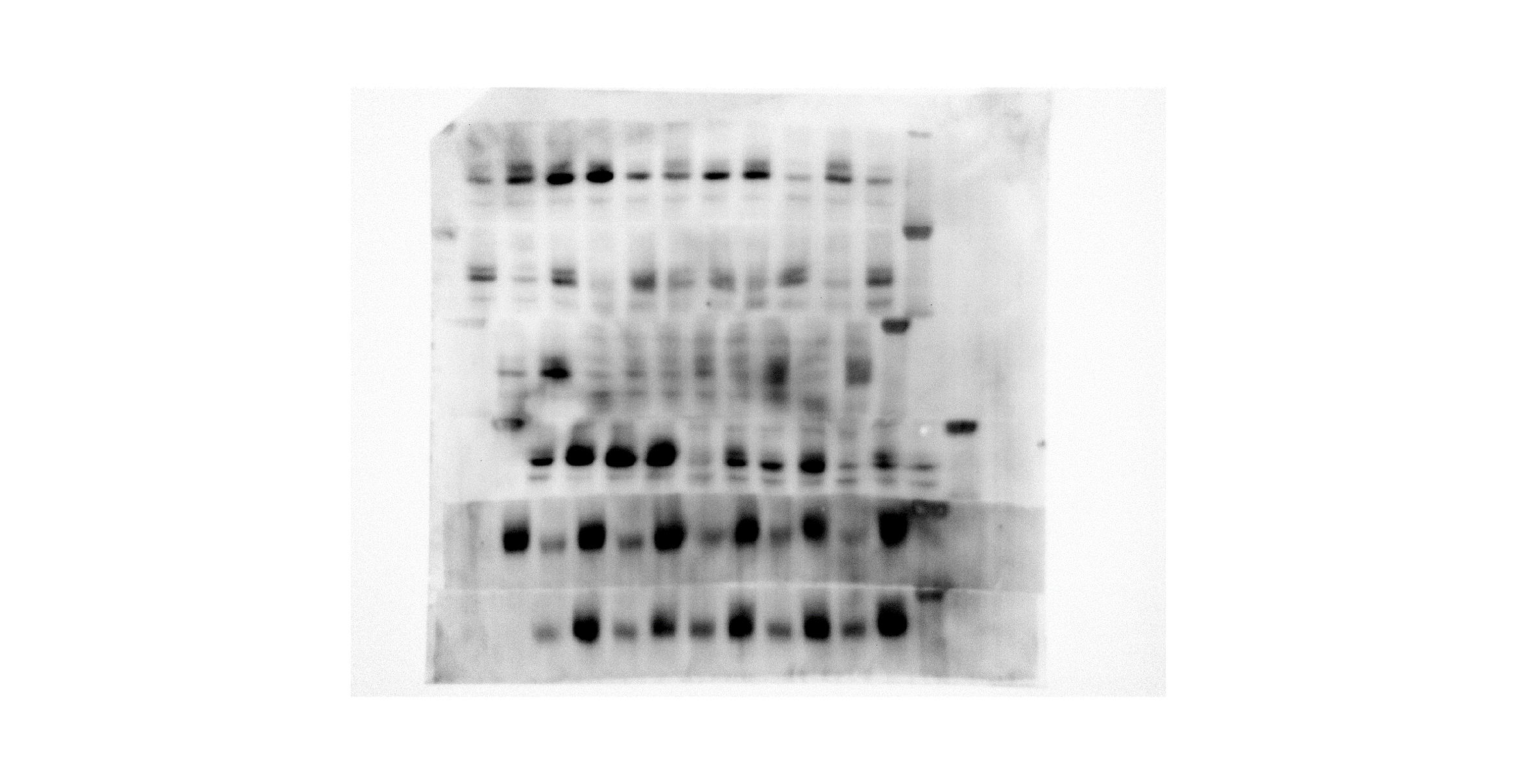

Supplement: Figure 4—figure supplement 1—source data 1. — Data used for quantification of Figure 4—figure supplement 1A–C, E–I and raw unedited blots for Figure 4—figure supplement 1A, B, D, I. [file elife-83338-fig4-figsupp1-data1.zip › Figure_4_-_supplement_1/Fig. 4 - supp. 1/Figure 4 - figure supplement 1 B AMPK 172 EDL+SOL.png]

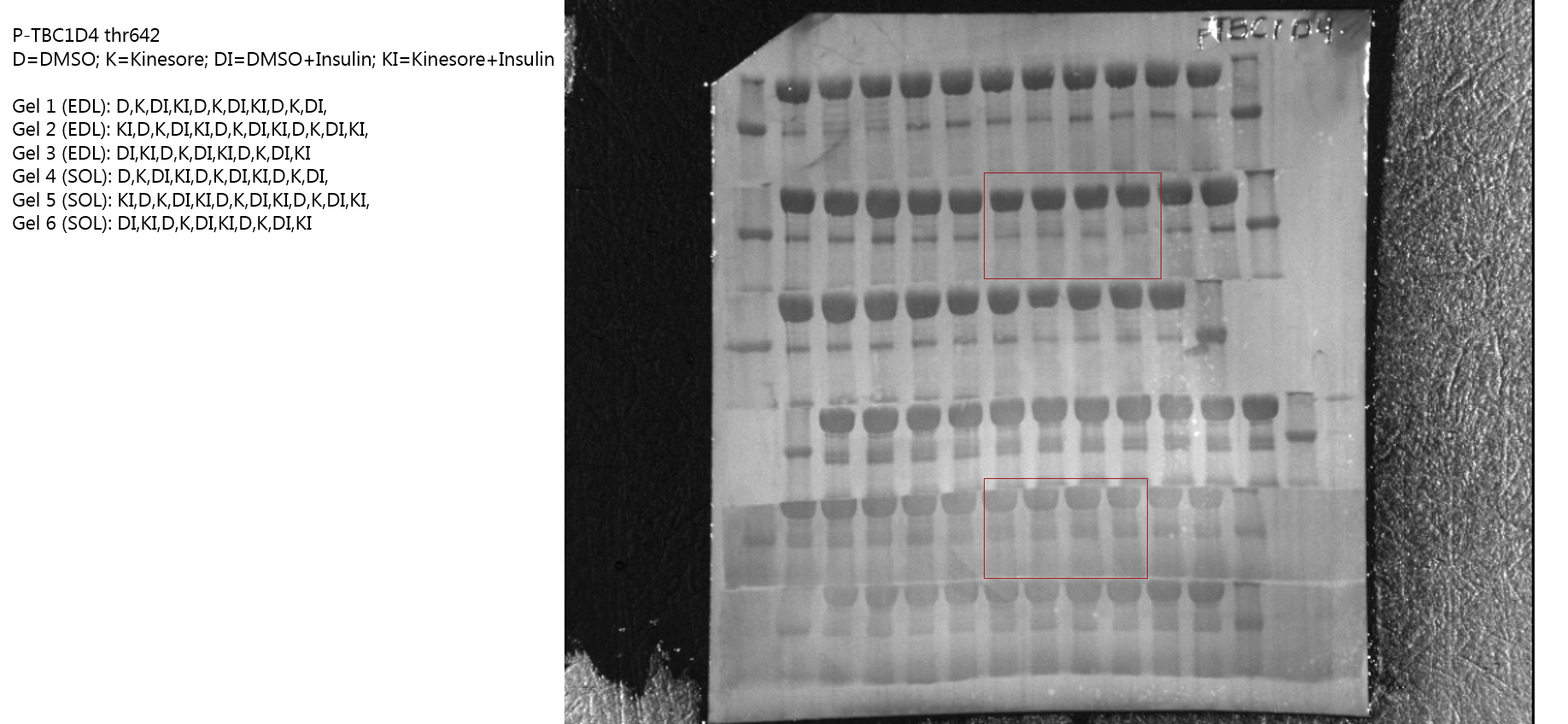

Supplement: Figure 4—figure supplement 1—source data 1. — Data used for quantification of Figure 4—figure supplement 1A–C, E–I and raw unedited blots for Figure 4—figure supplement 1A, B, D, I. [file elife-83338-fig4-figsupp1-data1.zip › Figure_4_-_supplement_1/Fig. 4 - supp. 1/Figure 4 - figure supplement 1 B coomassie EDL+SOL marked.png]

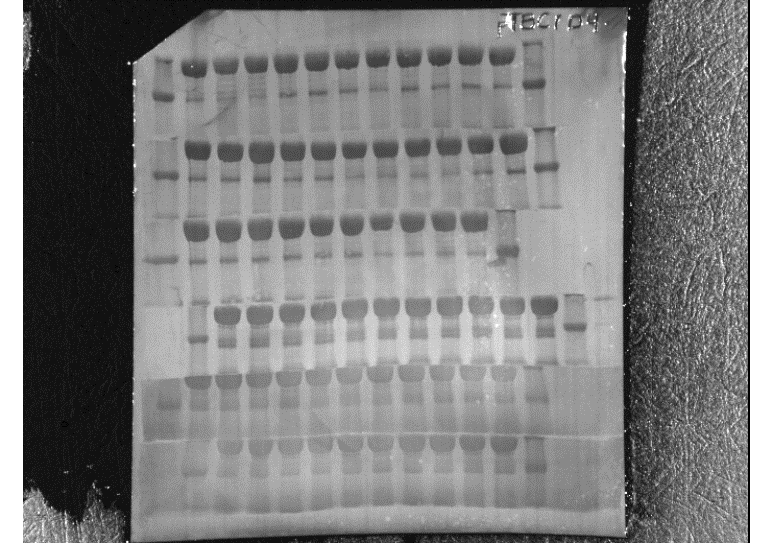

Supplement: Figure 4—figure supplement 1—source data 1. — Data used for quantification of Figure 4—figure supplement 1A–C, E–I and raw unedited blots for Figure 4—figure supplement 1A, B, D, I. [file elife-83338-fig4-figsupp1-data1.zip › Figure_4_-_supplement_1/Fig. 4 - supp. 1/Figure 4 - figure supplement 1 B coomassie EDL+SOL.png]

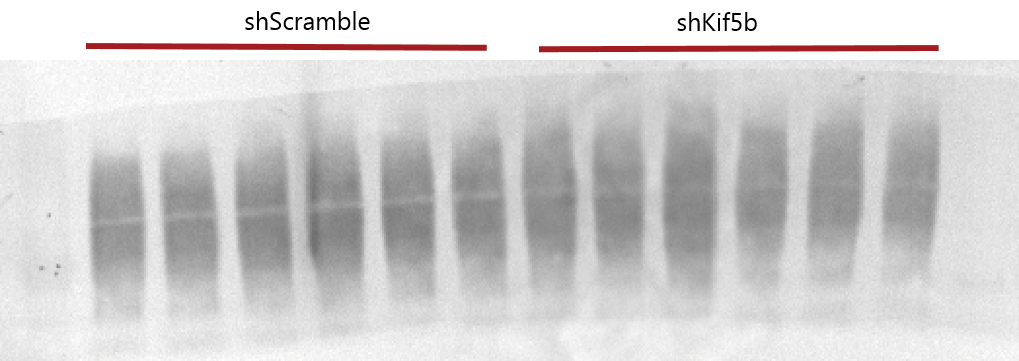

Supplement: Figure 4—figure supplement 1—source data 1. — Data used for quantification of Figure 4—figure supplement 1A–C, E–I and raw unedited blots for Figure 4—figure supplement 1A, B, D, I. [file elife-83338-fig4-figsupp1-data1.zip › Figure_4_-_supplement_1/Fig. 4 - supp. 1/Figure 4 - figure supplement 1 D coomassie membrane marked.png]

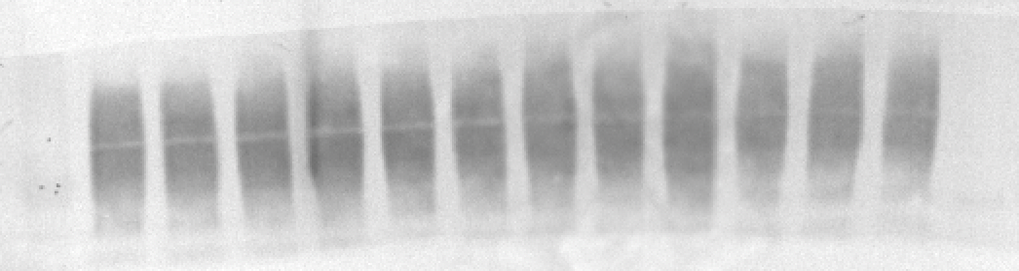

Supplement: Figure 4—figure supplement 1—source data 1. — Data used for quantification of Figure 4—figure supplement 1A–C, E–I and raw unedited blots for Figure 4—figure supplement 1A, B, D, I. [file elife-83338-fig4-figsupp1-data1.zip › Figure_4_-_supplement_1/Fig. 4 - supp. 1/Figure 4 - figure supplement 1 D coomassie membrane.png]

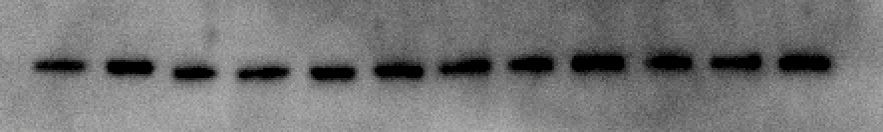

Supplement: Figure 4—figure supplement 1—source data 1. — Data used for quantification of Figure 4—figure supplement 1A–C, E–I and raw unedited blots for Figure 4—figure supplement 1A, B, D, I. [file elife-83338-fig4-figsupp1-data1.zip › Figure_4_-_supplement_1/Fig. 4 - supp. 1/Figure 4 - figure supplement 1 D GAPDH membrane .png]

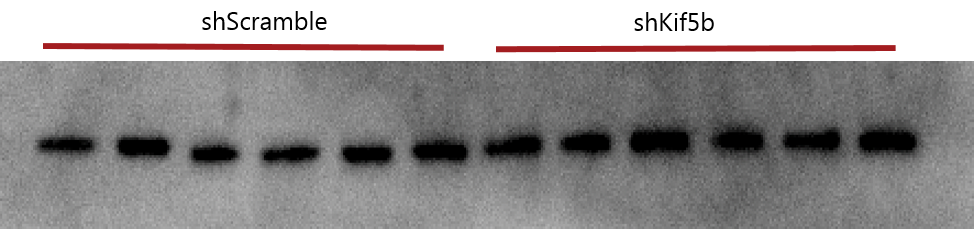

Supplement: Figure 4—figure supplement 1—source data 1. — Data used for quantification of Figure 4—figure supplement 1A–C, E–I and raw unedited blots for Figure 4—figure supplement 1A, B, D, I. [file elife-83338-fig4-figsupp1-data1.zip › Figure_4_-_supplement_1/Fig. 4 - supp. 1/Figure 4 - figure supplement 1 D GAPDH membrane marked .png]

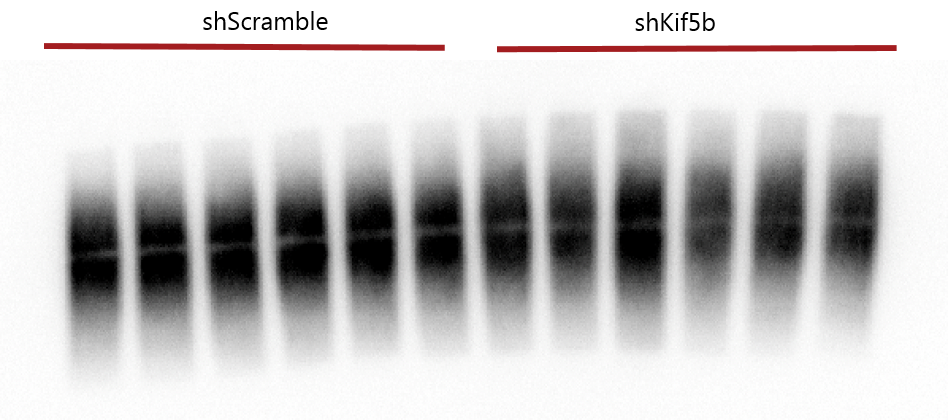

Supplement: Figure 4—figure supplement 1—source data 1. — Data used for quantification of Figure 4—figure supplement 1A–C, E–I and raw unedited blots for Figure 4—figure supplement 1A, B, D, I. [file elife-83338-fig4-figsupp1-data1.zip › Figure_4_-_supplement_1/Fig. 4 - supp. 1/Figure 4 - figure supplement 1 D GLUT4 membrane marked.png]

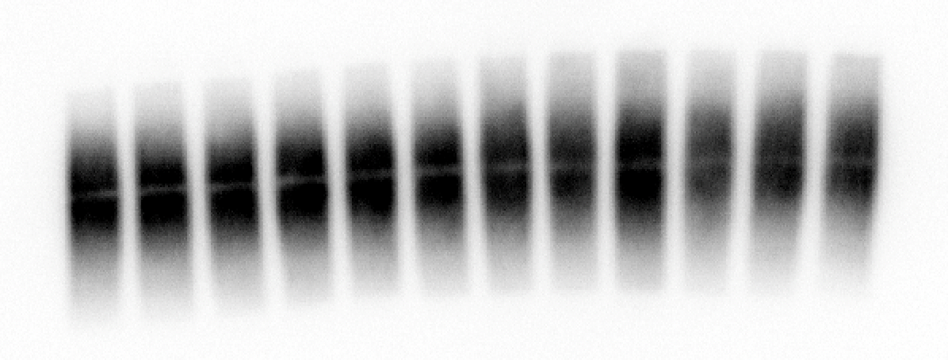

Supplement: Figure 4—figure supplement 1—source data 1. — Data used for quantification of Figure 4—figure supplement 1A–C, E–I and raw unedited blots for Figure 4—figure supplement 1A, B, D, I. [file elife-83338-fig4-figsupp1-data1.zip › Figure_4_-_supplement_1/Fig. 4 - supp. 1/Figure 4 - figure supplement 1 D GLUT4 membrane.png]

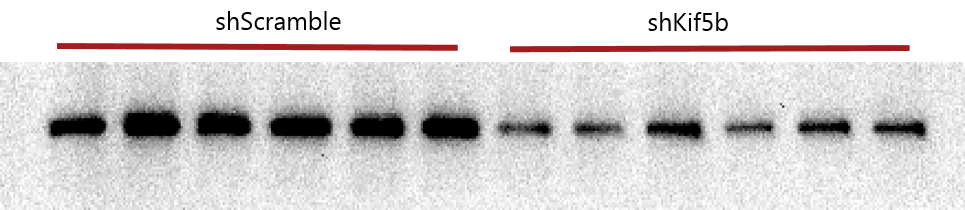

Supplement: Figure 4—figure supplement 1—source data 1. — Data used for quantification of Figure 4—figure supplement 1A–C, E–I and raw unedited blots for Figure 4—figure supplement 1A, B, D, I. [file elife-83338-fig4-figsupp1-data1.zip › Figure_4_-_supplement_1/Fig. 4 - supp. 1/Figure 4 - figure supplement 1 D KIF5B membrane marked.png]

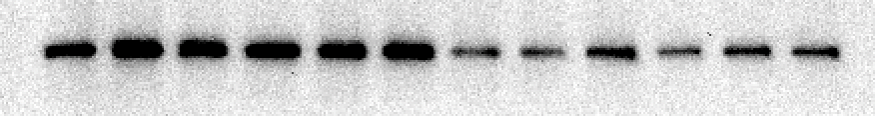

Supplement: Figure 4—figure supplement 1—source data 1. — Data used for quantification of Figure 4—figure supplement 1A–C, E–I and raw unedited blots for Figure 4—figure supplement 1A, B, D, I. [file elife-83338-fig4-figsupp1-data1.zip › Figure_4_-_supplement_1/Fig. 4 - supp. 1/Figure 4 - figure supplement 1 D KIF5B membrane.png]

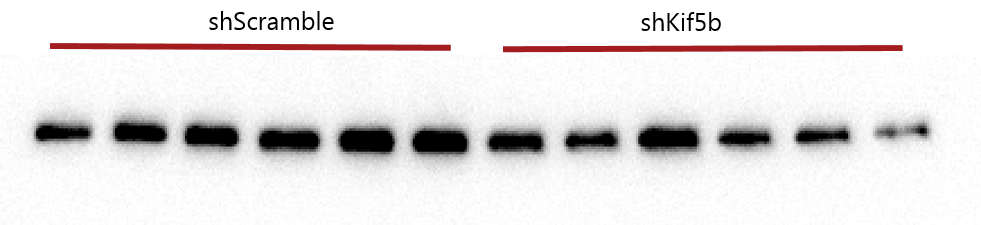

Supplement: Figure 4—figure supplement 1—source data 1. — Data used for quantification of Figure 4—figure supplement 1A–C, E–I and raw unedited blots for Figure 4—figure supplement 1A, B, D, I. [file elife-83338-fig4-figsupp1-data1.zip › Figure_4_-_supplement_1/Fig. 4 - supp. 1/Figure 4 - figure supplement 1 D tubulin membrane marked.png]

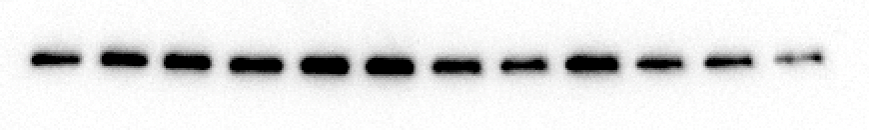

Supplement: Figure 4—figure supplement 1—source data 1. — Data used for quantification of Figure 4—figure supplement 1A–C, E–I and raw unedited blots for Figure 4—figure supplement 1A, B, D, I. [file elife-83338-fig4-figsupp1-data1.zip › Figure_4_-_supplement_1/Fig. 4 - supp. 1/Figure 4 - figure supplement 1 D tubulin membrane.png]

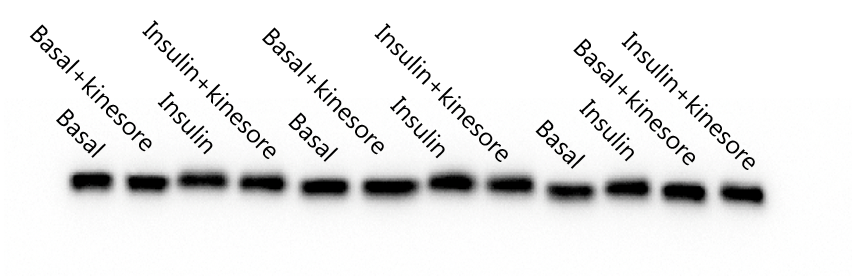

Supplement: Figure 4—figure supplement 1—source data 1. — Data used for quantification of Figure 4—figure supplement 1A–C, E–I and raw unedited blots for Figure 4—figure supplement 1A, B, D, I. [file elife-83338-fig4-figsupp1-data1.zip › Figure_4_-_supplement_1/Fig. 4 - supp. 1/Figure 4 - figure supplement 1 I Akt membrane marked.png]

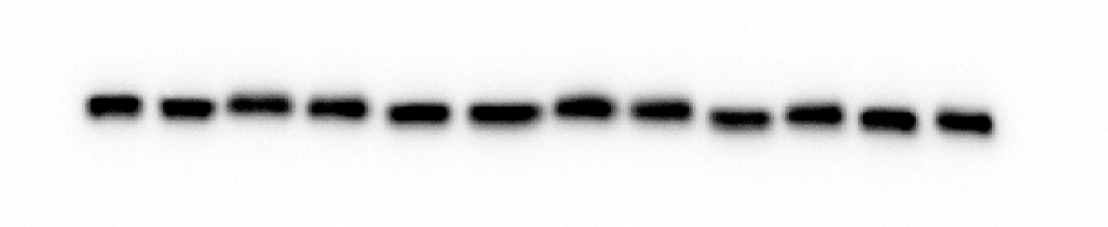

Supplement: Figure 4—figure supplement 1—source data 1. — Data used for quantification of Figure 4—figure supplement 1A–C, E–I and raw unedited blots for Figure 4—figure supplement 1A, B, D, I. [file elife-83338-fig4-figsupp1-data1.zip › Figure_4_-_supplement_1/Fig. 4 - supp. 1/Figure 4 - figure supplement 1 I Akt membrane.png]

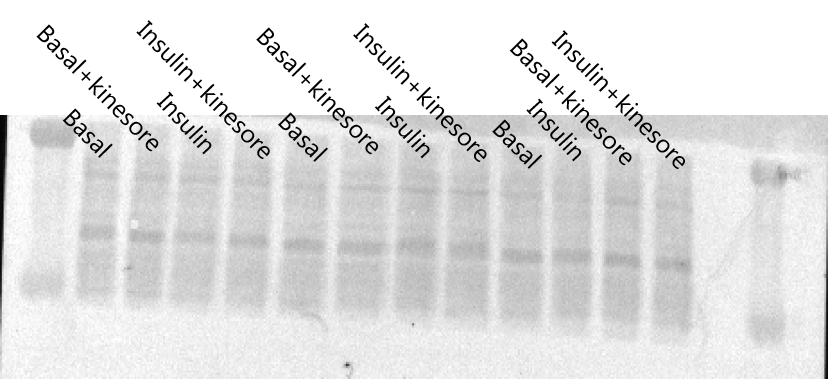

Supplement: Figure 4—figure supplement 1—source data 1. — Data used for quantification of Figure 4—figure supplement 1A–C, E–I and raw unedited blots for Figure 4—figure supplement 1A, B, D, I. [file elife-83338-fig4-figsupp1-data1.zip › Figure_4_-_supplement_1/Fig. 4 - supp. 1/Figure 4 - figure supplement 1 I Coomassie membrane marked.png]

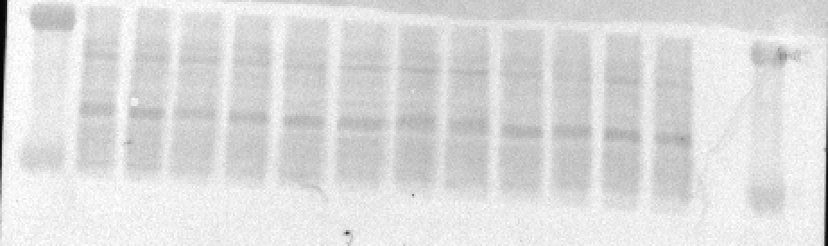

Supplement: Figure 4—figure supplement 1—source data 1. — Data used for quantification of Figure 4—figure supplement 1A–C, E–I and raw unedited blots for Figure 4—figure supplement 1A, B, D, I. [file elife-83338-fig4-figsupp1-data1.zip › Figure_4_-_supplement_1/Fig. 4 - supp. 1/Figure 4 - figure supplement 1 I Coomassie membrane.png]

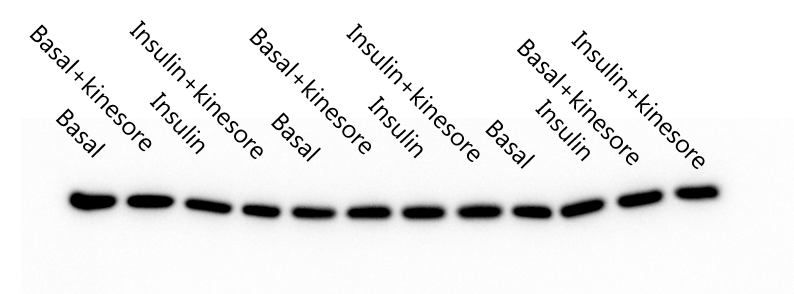

Supplement: Figure 4—figure supplement 1—source data 1. — Data used for quantification of Figure 4—figure supplement 1A–C, E–I and raw unedited blots for Figure 4—figure supplement 1A, B, D, I. [file elife-83338-fig4-figsupp1-data1.zip › Figure_4_-_supplement_1/Fig. 4 - supp. 1/Figure 4 - figure supplement 1 I GAPDH membrane marked.png]

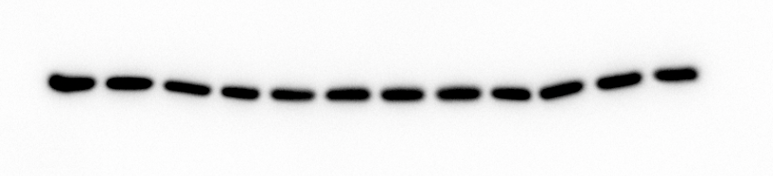

Supplement: Figure 4—figure supplement 1—source data 1. — Data used for quantification of Figure 4—figure supplement 1A–C, E–I and raw unedited blots for Figure 4—figure supplement 1A, B, D, I. [file elife-83338-fig4-figsupp1-data1.zip › Figure_4_-_supplement_1/Fig. 4 - supp. 1/Figure 4 - figure supplement 1 I GAPDH membrane.png]

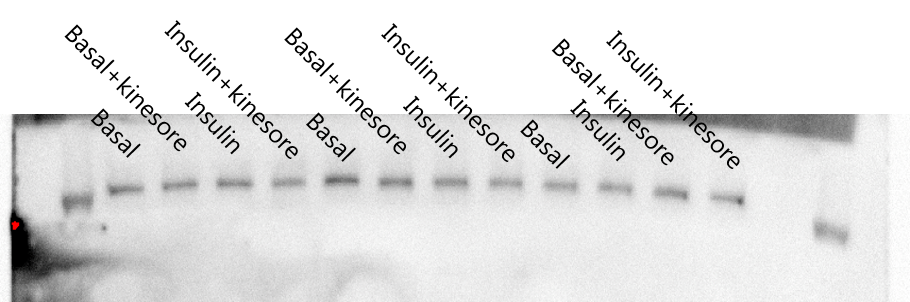

Supplement: Figure 4—figure supplement 1—source data 1. — Data used for quantification of Figure 4—figure supplement 1A–C, E–I and raw unedited blots for Figure 4—figure supplement 1A, B, D, I. [file elife-83338-fig4-figsupp1-data1.zip › Figure_4_-_supplement_1/Fig. 4 - supp. 1/Figure 4 - figure supplement 1 I p-ACC 222 membrane marked.png]

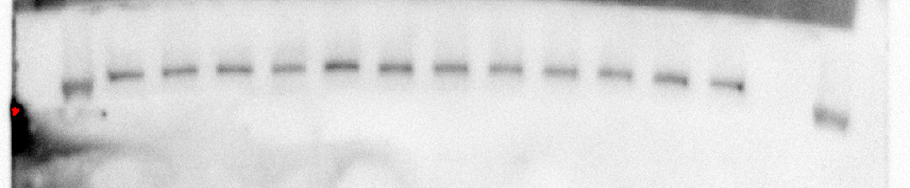

Supplement: Figure 4—figure supplement 1—source data 1. — Data used for quantification of Figure 4—figure supplement 1A–C, E–I and raw unedited blots for Figure 4—figure supplement 1A, B, D, I. [file elife-83338-fig4-figsupp1-data1.zip › Figure_4_-_supplement_1/Fig. 4 - supp. 1/Figure 4 - figure supplement 1 I p-ACC 222 membrane.png]

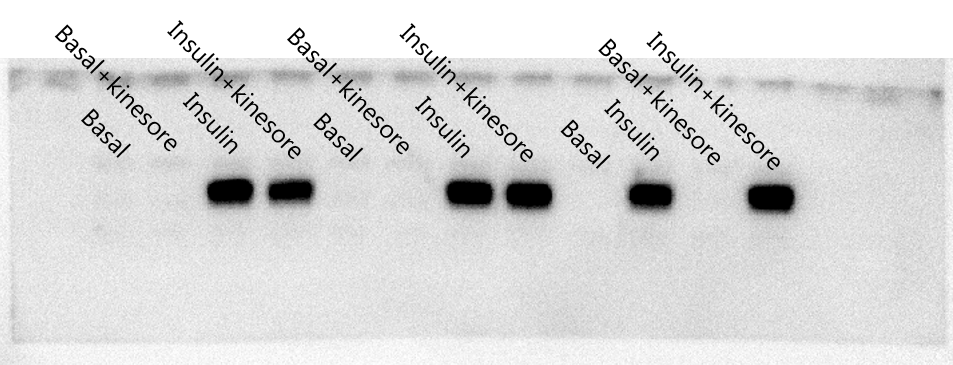

Supplement: Figure 4—figure supplement 1—source data 1. — Data used for quantification of Figure 4—figure supplement 1A–C, E–I and raw unedited blots for Figure 4—figure supplement 1A, B, D, I. [file elife-83338-fig4-figsupp1-data1.zip › Figure_4_-_supplement_1/Fig. 4 - supp. 1/Figure 4 - figure supplement 1 I p-Akt 308 membrane marked.png]

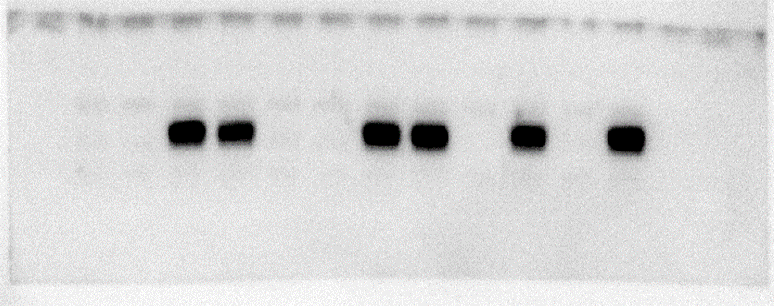

Supplement: Figure 4—figure supplement 1—source data 1. — Data used for quantification of Figure 4—figure supplement 1A–C, E–I and raw unedited blots for Figure 4—figure supplement 1A, B, D, I. [file elife-83338-fig4-figsupp1-data1.zip › Figure_4_-_supplement_1/Fig. 4 - supp. 1/Figure 4 - figure supplement 1 I p-Akt 308 membrane.png]

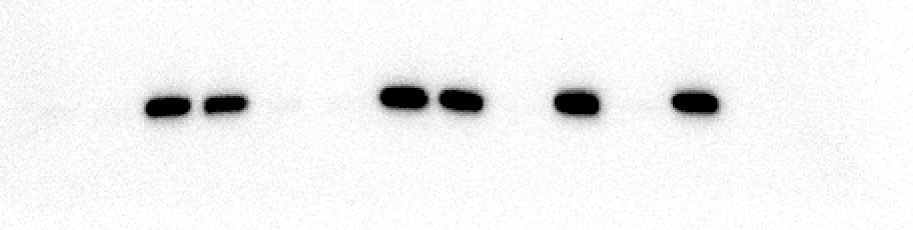

Supplement: Figure 4—figure supplement 1—source data 1. — Data used for quantification of Figure 4—figure supplement 1A–C, E–I and raw unedited blots for Figure 4—figure supplement 1A, B, D, I. [file elife-83338-fig4-figsupp1-data1.zip › Figure_4_-_supplement_1/Fig. 4 - supp. 1/Figure 4 - figure supplement 1 I p-Akt 473 membrane marked.png]

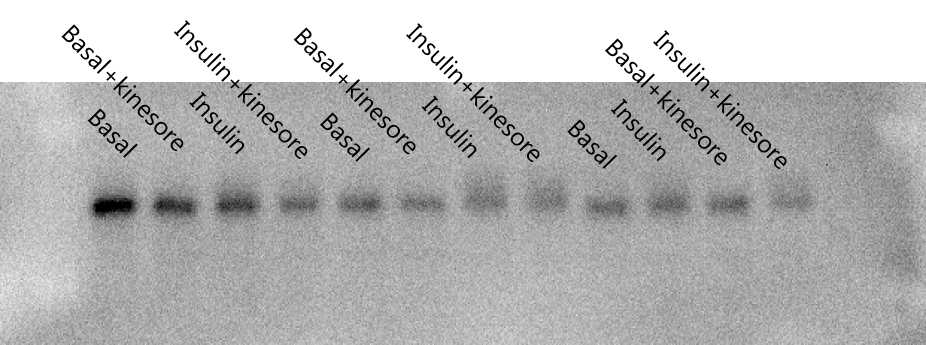

Supplement: Figure 4—figure supplement 1—source data 1. — Data used for quantification of Figure 4—figure supplement 1A–C, E–I and raw unedited blots for Figure 4—figure supplement 1A, B, D, I. [file elife-83338-fig4-figsupp1-data1.zip › Figure_4_-_supplement_1/Fig. 4 - supp. 1/Figure 4 - figure supplement 1 I p-AMPK 172 membrane marked.png]

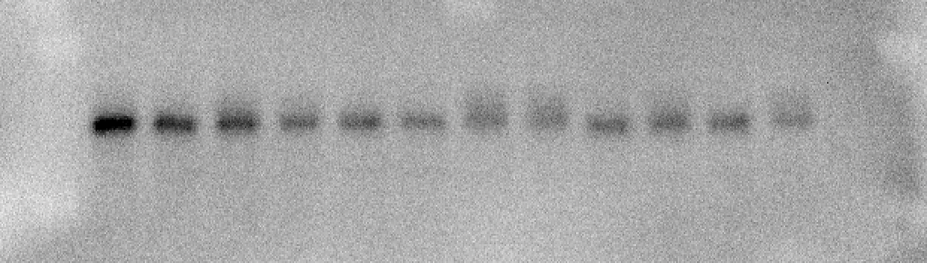

Supplement: Figure 4—figure supplement 1—source data 1. — Data used for quantification of Figure 4—figure supplement 1A–C, E–I and raw unedited blots for Figure 4—figure supplement 1A, B, D, I. [file elife-83338-fig4-figsupp1-data1.zip › Figure_4_-_supplement_1/Fig. 4 - supp. 1/Figure 4 - figure supplement 1 I p-AMPK 172 membrane.png]

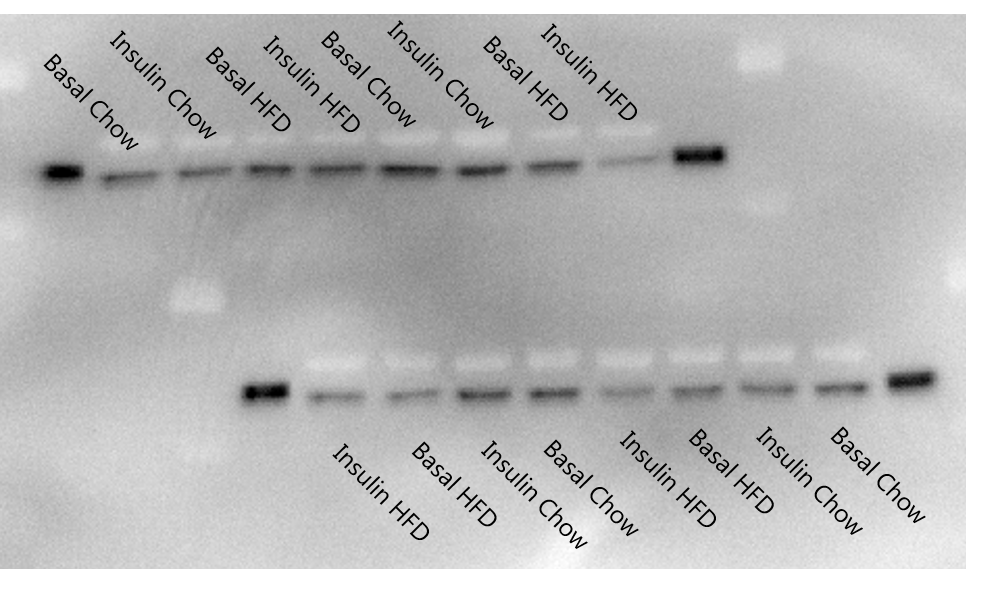

Supplement: Figure 5—figure supplement 1—source data 1. — Data used for quantification of Figure 5—figure supplement 1A–F and H–J and raw unedited blots for Figure 5—figure supplement 1A, D. [file elife-83338-fig5-figsupp1-data1.zip › Fig. 5 - supp. 1/Figure 5 - figure supplement 1 D Akt membrane marked.png]

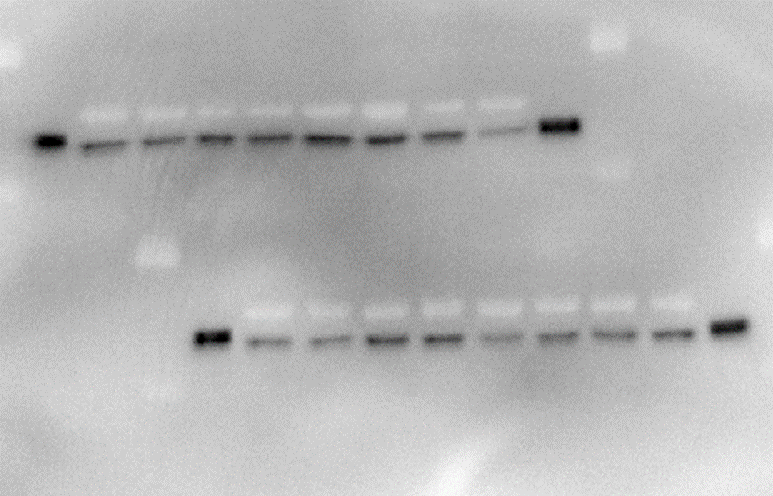

Supplement: Figure 5—figure supplement 1—source data 1. — Data used for quantification of Figure 5—figure supplement 1A–F and H–J and raw unedited blots for Figure 5—figure supplement 1A, D. [file elife-83338-fig5-figsupp1-data1.zip › Fig. 5 - supp. 1/Figure 5 - figure supplement 1 D Akt membrane.png]

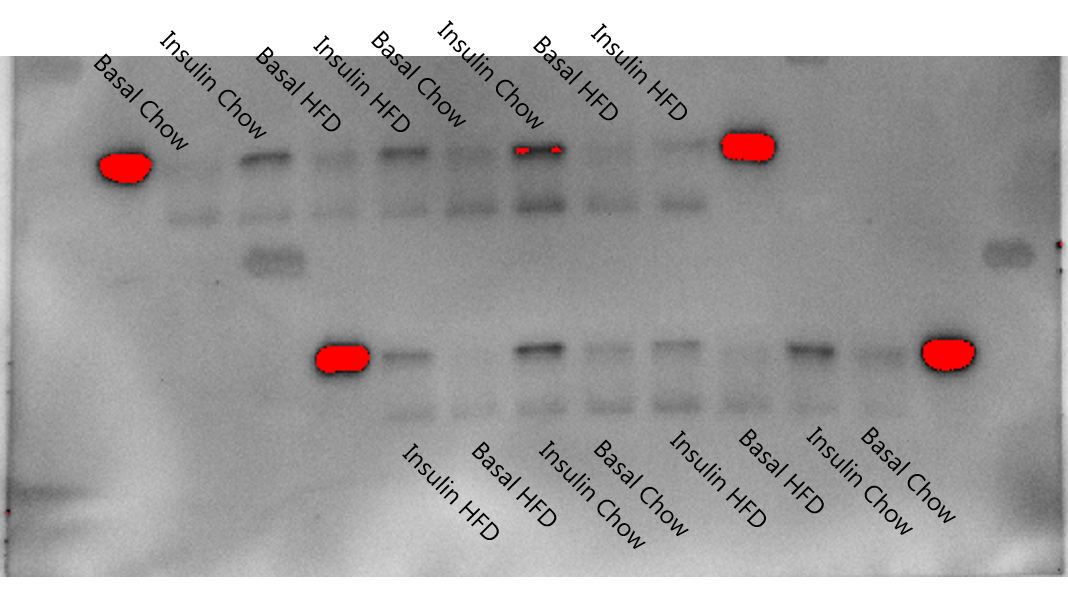

Supplement: Figure 5—figure supplement 1—source data 1. — Data used for quantification of Figure 5—figure supplement 1A–F and H–J and raw unedited blots for Figure 5—figure supplement 1A, D. [file elife-83338-fig5-figsupp1-data1.zip › Fig. 5 - supp. 1/Figure 5 - figure supplement 1 D p-Akt 308 membrane marked.png]

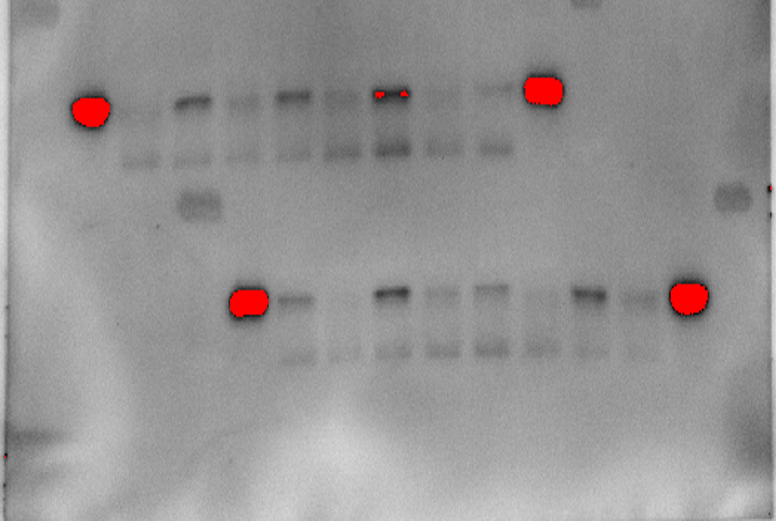

Supplement: Figure 5—figure supplement 1—source data 1. — Data used for quantification of Figure 5—figure supplement 1A–F and H–J and raw unedited blots for Figure 5—figure supplement 1A, D. [file elife-83338-fig5-figsupp1-data1.zip › Fig. 5 - supp. 1/Figure 5 - figure supplement 1 D p-Akt 308 membrane.png]

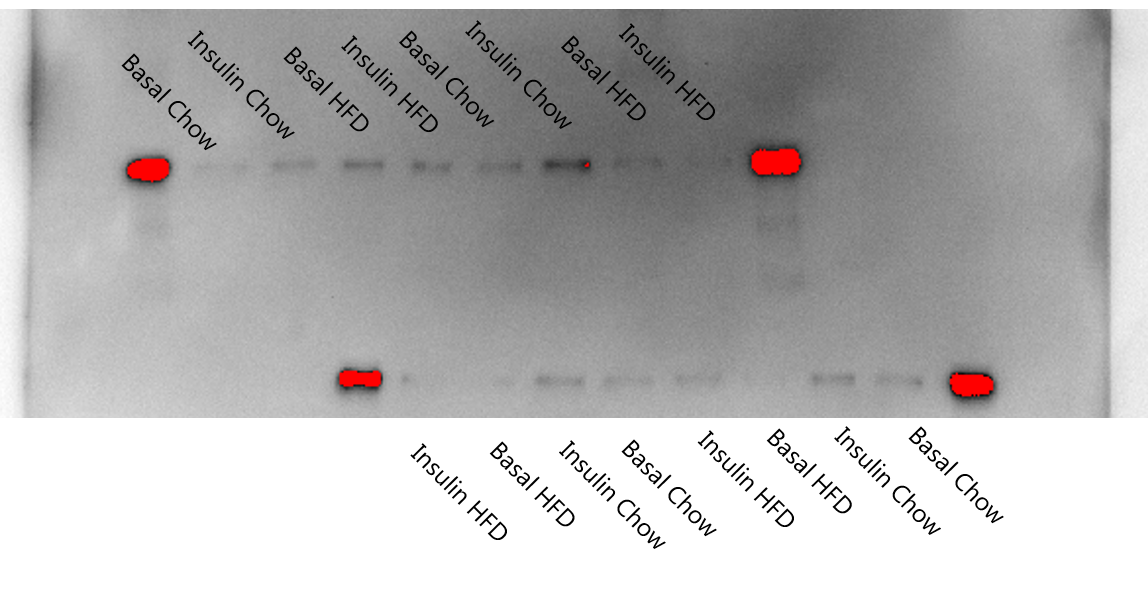

Supplement: Figure 5—figure supplement 1—source data 1. — Data used for quantification of Figure 5—figure supplement 1A–F and H–J and raw unedited blots for Figure 5—figure supplement 1A, D. [file elife-83338-fig5-figsupp1-data1.zip › Fig. 5 - supp. 1/Figure 5 - figure supplement 1 D p-tbc1d4 642 membrane marked.png]

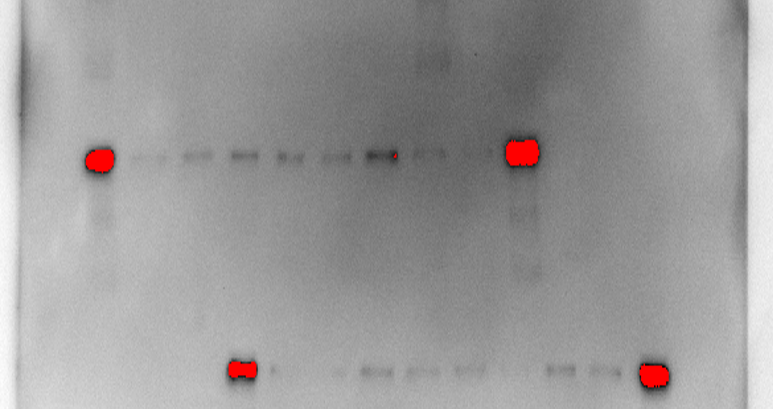

Supplement: Figure 5—figure supplement 1—source data 1. — Data used for quantification of Figure 5—figure supplement 1A–F and H–J and raw unedited blots for Figure 5—figure supplement 1A, D. [file elife-83338-fig5-figsupp1-data1.zip › Fig. 5 - supp. 1/Figure 5 - figure supplement 1 D p-tbc1d4 642 membrane.png]

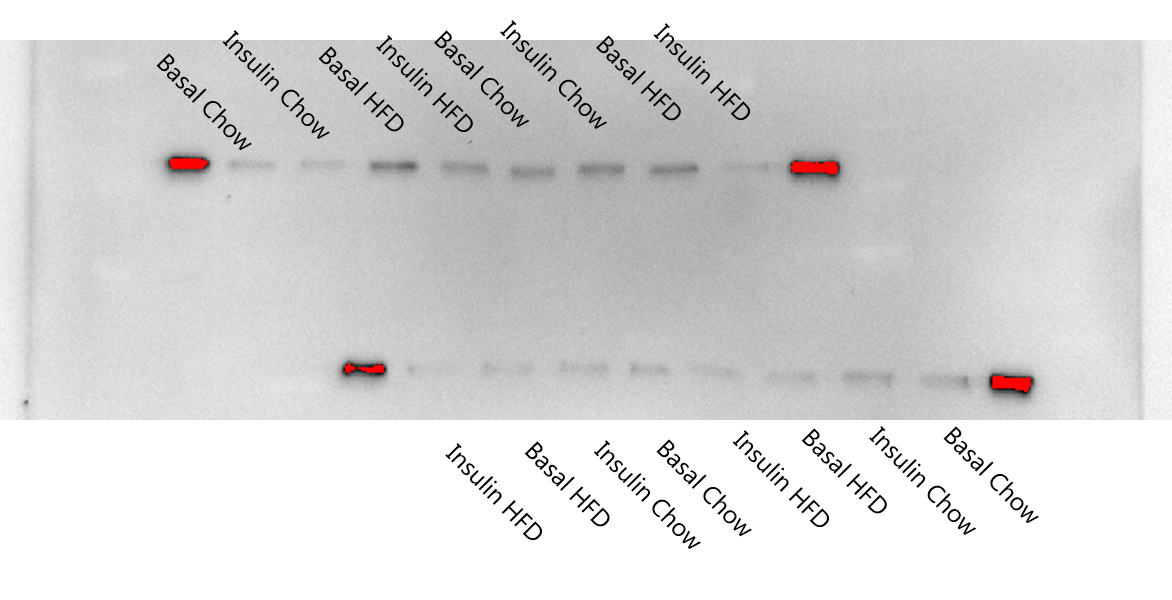

Supplement: Figure 5—figure supplement 1—source data 1. — Data used for quantification of Figure 5—figure supplement 1A–F and H–J and raw unedited blots for Figure 5—figure supplement 1A, D. [file elife-83338-fig5-figsupp1-data1.zip › Fig. 5 - supp. 1/Figure 5 - figure supplement 1 D tbc1d4 membrane marked.png]

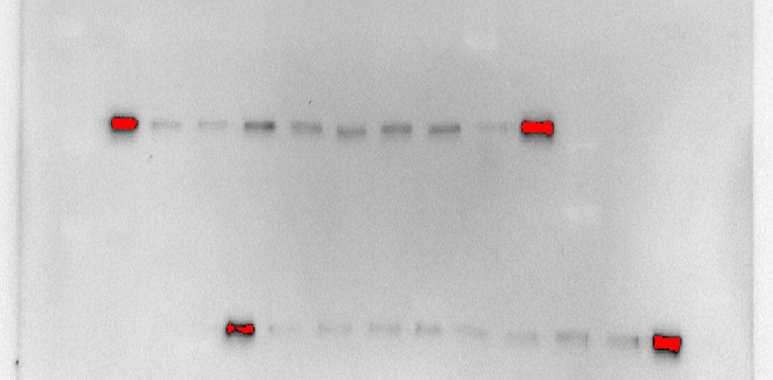

Supplement: Figure 5—figure supplement 1—source data 1. — Data used for quantification of Figure 5—figure supplement 1A–F and H–J and raw unedited blots for Figure 5—figure supplement 1A, D. [file elife-83338-fig5-figsupp1-data1.zip › Fig. 5 - supp. 1/Figure 5 - figure supplement 1 D tbc1d4 membrane.png]

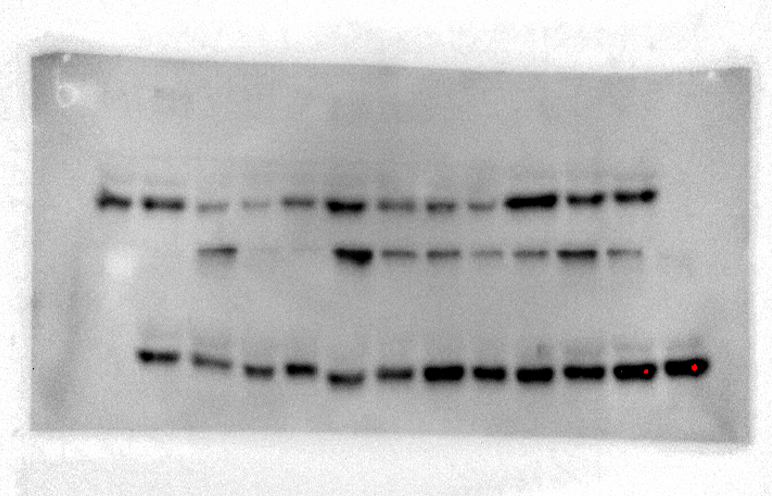

Supplement: Figure 5—figure supplement 1—source data 1. — Data used for quantification of Figure 5—figure supplement 1A–F and H–J and raw unedited blots for Figure 5—figure supplement 1A, D. [file elife-83338-fig5-figsupp1-data1.zip › Fig. 5 - supp. 1/Figure 5 - supplement 1 A Akt membrane .png]

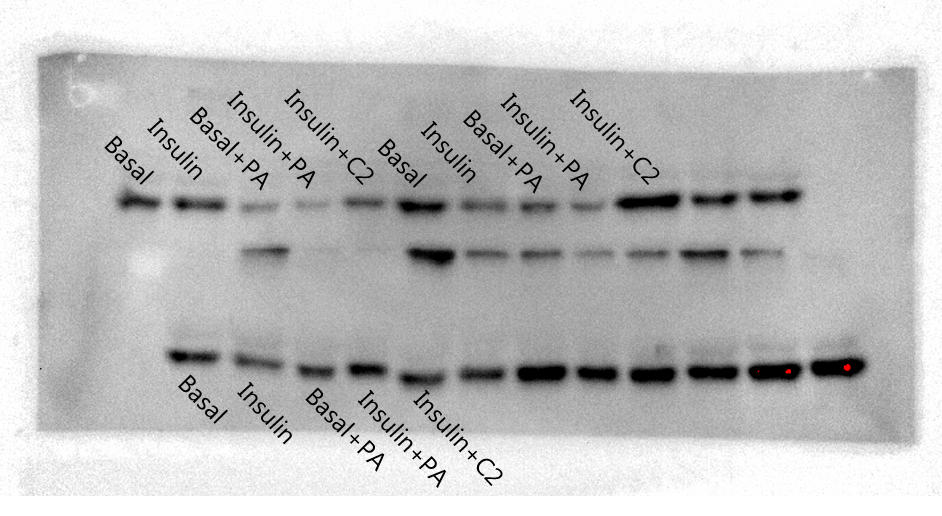

Supplement: Figure 5—figure supplement 1—source data 1. — Data used for quantification of Figure 5—figure supplement 1A–F and H–J and raw unedited blots for Figure 5—figure supplement 1A, D. [file elife-83338-fig5-figsupp1-data1.zip › Fig. 5 - supp. 1/Figure 5 - supplement 1 A Akt membrane marked .png]

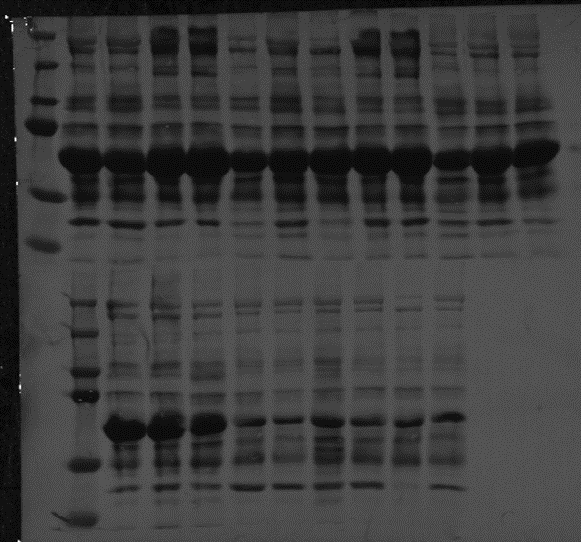

Supplement: Figure 5—figure supplement 1—source data 1. — Data used for quantification of Figure 5—figure supplement 1A–F and H–J and raw unedited blots for Figure 5—figure supplement 1A, D. [file elife-83338-fig5-figsupp1-data1.zip › Fig. 5 - supp. 1/Figure 5 - supplement 1 A coomassie membrane .png]

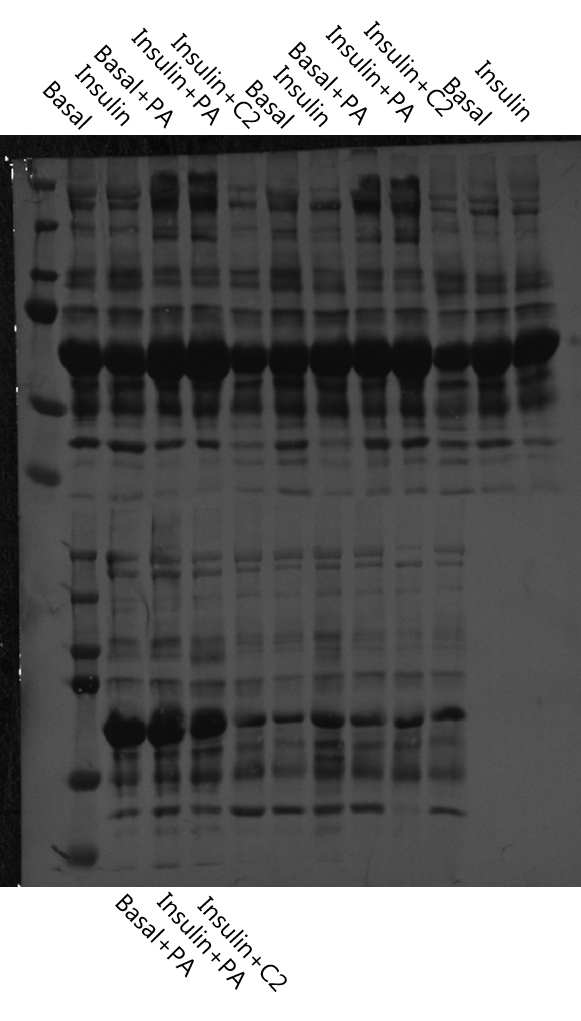

Supplement: Figure 5—figure supplement 1—source data 1. — Data used for quantification of Figure 5—figure supplement 1A–F and H–J and raw unedited blots for Figure 5—figure supplement 1A, D. [file elife-83338-fig5-figsupp1-data1.zip › Fig. 5 - supp. 1/Figure 5 - supplement 1 A coomassie membrane marked.png]

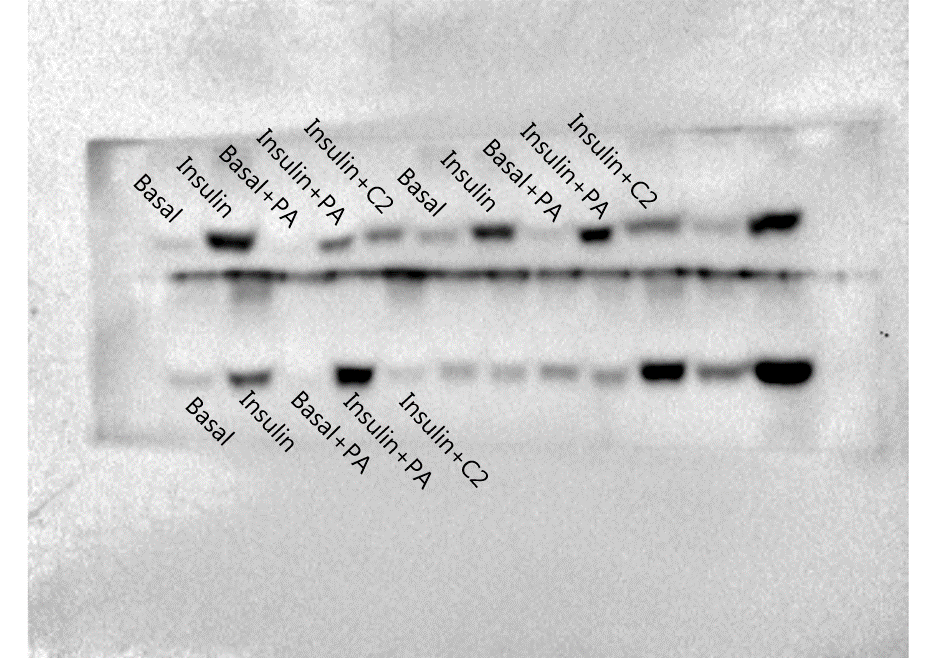

Supplement: Figure 5—figure supplement 1—source data 1. — Data used for quantification of Figure 5—figure supplement 1A–F and H–J and raw unedited blots for Figure 5—figure supplement 1A, D. [file elife-83338-fig5-figsupp1-data1.zip › Fig. 5 - supp. 1/Figure 5 - supplement 1 A p-Akt 308 membrane marked .png]

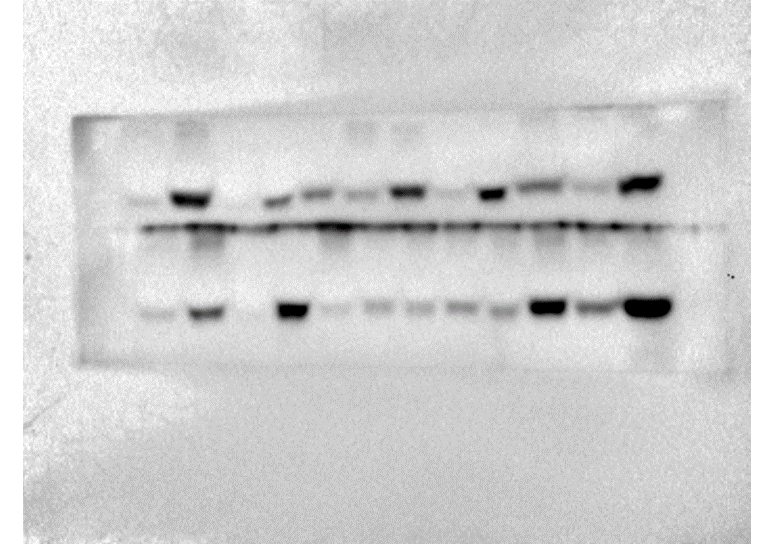

Supplement: Figure 5—figure supplement 1—source data 1. — Data used for quantification of Figure 5—figure supplement 1A–F and H–J and raw unedited blots for Figure 5—figure supplement 1A, D. [file elife-83338-fig5-figsupp1-data1.zip › Fig. 5 - supp. 1/Figure 5 - supplement 1 A p-Akt 308 membrane.png]
